# Multilevel analysis of individual heterogeneity and discriminatory accuracy (MAIHDA) to understand how obesity risk varies according to multiple lifestyle behavior recommendations

**DOI:** 10.1038/s41366-025-02010-1

**Published:** 2026-01-07

**Authors:** Ansuman Swain, Natalie Pearson, Scott A. Willis, William Johnson

**Affiliations:** 1 National Centre for Sport and Exercise Medicine, School of Sport, Exercise and Health Sciences, Loughborough University, Loughborough, United Kingdom; 2 NIHR Leicester Biomedical Research Centre, University Hospitals of Leicester NHS Trust and the University of Leicester, Leicester, United Kingdom

**Keywords:** Epidemiology, Obesity, Epidemiology, Lifestyle modification

## Abstract

**Background:**

The combined and interactive effects of multiple lifestyle behaviours on obesity risk are not well understood. We used Multilevel Analysis of Individual Heterogeneity and Discriminatory Accuracy (MAIHDA) to examine how adherence to public health recommendations for five lifestyle behaviours affects BMI and obesity risk.

**Methods:**

The sample included 139,540 men and 125,455 women from the UK Biobank. We categorized fruit and vegetable intake, physical activity, sleep duration and alcohol intake as binary variables (meeting vs. not meeting guidelines), and smoking status into three categories (previous, current, never). These categories were combined to form 48 unique strata, representing all possible combinations of the five behaviours. Linear and binary logistic MAIHDA models were used, with individuals nested within strata, and BMI and obesity status (obesity vs. normal weight) as outcomes. Three models were employed: Model 1 (null), Model 2 (with fixed effects for lifestyle behaviours), and Model 3 (with confounders and fixed effects). Variance Partition Coefficient (VPC), Proportional Change in Variance (PCV), and predicted BMI and obesity risk were estimated.

**Results:**

For both sexes, strata with the lowest obesity risk were associated with meeting most recommendations, while strata with the highest risk were linked to meeting few. Logistic Model 1 VPCs revealed 7% of variance in obesity risk among males and 5% among females was explained by between-strata differences. In Model 3, VPCs attenuated to 0.5% among males and 0.1% among females, suggesting differences in obesity risk were largely additive effects. PCVs from Model 3 also indicated primarily additive rather than interactive effects. Results were similar for BMI in the linear models.

**Conclusions:**

Using a novel statistical approach, this study shows that additive effects of multiple lifestyle behaviours predominantly explain differences in BMI and obesity risk. Meeting more public health lifestyle recommendations is important in mitigating obesity risk.

## Introduction

Obesity is a major public health issue and an established risk factor for many other chronic health conditions such as type 2 diabetes, coronary heart disease, neurological disorders, and multiple cancers. In the UK, 26% of the adult population were living with obesity in 2021, with projections suggesting this number will rise to almost 37% by 2034 [1, 2]. The estimated annual cost of obesity to the UK’s National Health Service (NHS) is £6.5 billion [3].

Obesity is associated with many interrelated lifestyle behaviours including a sedentary and physically inactive lifestyle, and excessive caloric intake [1, 4–8]. The World Health Organisation recommends at least 150 min of moderate- or 75 min of vigorous-intensity physical activity weekly to reduce obesity risk [9–11]. Likewise, evidence suggests that consuming at least five portions of fruits and vegetables daily is associated with lower BMI and obesity risk [12, 13]. Other modifiable behaviours, such as sleep duration, alcohol intake, and smoking, also contribute to obesity risk [13–18]. Both short and long sleep durations outside the NHS recommended 7–9 h/day are associated with greater BMI and obesity risk [16, 17, 19, 20]. Furthermore, exceeding the NHS alcohol intake guidelines (no more than 14 units per week) and being an ex-smoker, are linked to higher BMI [15, 16, 21–25].

While the associations between individual lifestyle behaviours and obesity risk are well studied, these behaviours do not occur in isolation. There may be important interactions (i.e. multiplicative effects) between behaviours which are not captured when studied separately [26–28]. For instance, the weight gain associated with smoking cessation may be mitigated by regular physical activity [27]. In another study, Kaufman et al., highlighted the synergistic effect of smoking and sedentary behaviour on obesity risk [29]. Nurwanti et al. have also demonstrated that the positive association between sedentary behaviour and obesity risk is amplified by greater carbohydrate intake [30]. Additionally, Kim et al. further illustrated that obesity risk was highest among individuals who reported insufficient sleep and frequent binge alcohol consumption [31]. Furthermore, there is evidence suggesting that when behaviours are examined in isolation, the impact on obesity risk is not fully explained compared to when studying multiple or synergistic effects of behaviours [32, 33]. The combined and interactive associations of multiple (>2) lifestyle behaviours with BMI and obesity risk remain inadequately understood [27, 28, 34]. This is because of challenges in modelling health outcomes based on multiple lifestyle behaviours, while accounting for the high number of possible interactions. While various approaches exist to address this, one such method is Latent Class Analysis (LCA), which categorizes individuals into distinct subgroups based on behaviour patterns [33, 35]. However, LCA and similar approaches may still fail to fully capture the complex, individual-level interactions between behaviours that influence obesity risk, as they primarily focus on grouping individuals rather than modelling the dynamic interrelationships between multiple behaviours.

Multilevel analysis of individual heterogeneity and discriminatory accuracy (MAIHDA) is a method predominantly used to investigate intersectional inequalities [36, 37]. It involves creating intersectional groups or strata (e.g. one stratum might be Black ethnicity, female sex, aged 40–60 years, high education, living in a deprived area) and fitting a multilevel model, with individuals at level one nested within strata at level two. This approach addresses many limitations of conventional methods (e.g. a standard regression model with all possible interactions), with advantages including model parsimony, precision weighted estimates, and accurate estimates for strata with small sample sizes [34, 38]. MAIHDA efficiently allows the researcher to 1) estimate the predicted outcome for each user-defined group/stratum of interest, 2) compare between strata vs. between individual within strata variation, and 3) separate additive vs. multiplicative effects. Extensive details about the MAIHDA method have been described elsewhere [36, 38, 39].

While MAIHDA has gained serious traction in social epidemiology to study intersectional inequalities, it has not yet been used widely in other fields. We intended to demonstrate for the first time how MAIHDA can be used in behavioural obesity epidemiology. This study aimed to investigate the combined, interactive associations of five lifestyle behaviours (diet, physical activity, sleeping, smoking, and alcohol intake) with BMI and obesity risk.

## Research design and methods

### Study and sample

We conducted a cross-sectional study using baseline data from the UK Biobank – a large, prospective cohort study of over 500,000 adults aged 40–69 years between 2006 and 2010. Written informed consent was obtained from all participants. More details on the UK Biobank study are available online [40–42]. This research was part of project number 80843.

After excluding 237,361 participants due to missing data (233,866 for lifestyle behaviours, 2506 for confounders, and 989 for BMI), 264,995 participants (139,540 males, 125,455 females) were selected for the final analysis (Supplementary Fig. 1).

### Lifestyle variables

At the UK Biobank baseline assessment visit, lifestyle variables were assessed through a touchscreen questionnaire. Four of the five lifestyle variables were re-coded according to meeting vs. not meeting the following UK national public health guidelines: sleep duration (7–9 h per day), fruit and vegetable intake (at least 5 portions per day), alcohol intake (no more than 14 units per week), and physical activity (at least 150 min of moderate- or 75 min of vigorous-intensity activity weekly). The fifth lifestyle variable, smoking status, was coded as current, previous, and never smoker. More details on the lifestyle variables and recoding are in Supplementary Table 1.

### Lifestyle strata

Forty-eight unique groups or strata (i.e. 2 × 2 × 2 × 2 × 3) were created based on combinations of these lifestyle behaviours. Each stratum was assigned a 5-digit number, with each digit representing whether the participant met (1) or did not meet (0) the relevant lifestyle behaviour recommendation. Smoking was coded as current (1), previous (0), and never (2). For example, a 5-digit number of 01112 would represent participants who did not meet the sleep duration guideline (first digit = 0), met the fruit and vegetable intake guideline (second digit = 1), met the alcohol intake guideline (third digit = 1), met the physical activity guideline (fourth digit = 1), and were never smokers (fifth digit = 2). Further details on how the strata were constructed are provided in Supplementary Fig. 2.

By stratifying participants into these lifestyle profile groups, the MAIHDA approach enables us to quantify whether there is any additional strata-level effect beyond the sum of individual behaviours. This also allows us to understand whether certain lifestyle combinations have additional influence on BMI, and obesity risk compared to considering the behaviours individually.

### Outcomes

Standing height and weight of participants were measured by trained staff using a non-stretchable sprung tape measure and standardized scales [39, 40]. The outcomes of interest were BMI (kg/m^2^) and weight status (underweight <18.5 kg/m^2^, normal weight 18.5–24.9 kg/m^2^, overweight 25.0–29.9 kg/m^2^, and obesity >30 kg/m^2^) [39]. The underweight category was excluded due to low participant numbers (*N* = 1149).

### Confounders

Age, ethnicity, and socioeconomic position were considered as potential confounders. Age and ethnicity were self-reported at the time of recruitment. Ethnicity was self-reported and categorized as white and non-white (mixed, Asian or Asian British, Black or Black British, and other). Indicators of socioeconomic position included the Townsend Deprivation Index (TDI), where participants were asked to report their home postcode, and self-reported employment status (currently employed vs. retired or unemployed).

### Statistical analysis

All analyses were stratified by sex. Descriptive statistics were utilised to describe the participants and variables of interest, and linear and binary logistic MAIHDA models were fit to BMI and weight status, respectively. To evaluate potential multicollinearity among the predictors, we examined pairwise correlations and calculated the variance inflation factors (VIFs) for all lifestyle behaviours and confounders, separately for males and females.

Although we initially attempted to use a multinomial logistic model to examine both overweight and obesity status in one model, this approach resulted in convergence issues with the models, prompting us to use two separate binary logistic models instead.

Three models were fit for each outcome:

### Model 1: Null model

The first model included only a random intercept with no predictor variables.

$${\rm{Linear}}:{{bmi}}_{{ij}}={\beta }_{0{ij}}$$

$${\beta }_{0{ij}}={\beta }_{0}+{u}_{0j}+{e}_{0{ij}}$$

where ${y}_{{ij}}$ denotes the BMI for individual ${i}$ in stratum $j$, ${\beta }_{0}$ represents the precision-weighted grand mean BMI, ${u}_{j}$ signifies the stratum random effect, and ${e}_{{ij}}$ indicates the deviation of the observed BMI for individual $i$ in stratum $j$ from the stratum mean BMI estimate.

$${Logistic}:{logit}\left({\pi }_{j}\right)=\log \left(\frac{{\pi }_{j}}{1-{\pi }_{j}}\right)={\beta }_{0}+{u}_{j}$$

where ${logit}\left({\pi }_{j}\right)$ denotes the log odds of obesity (vs. normal weight), $\beta$0 represents the precision-weighted grand mean, and ${u}_{j}$ signifies the stratum random effect.

### Model 2: Main effects model

In the second models, the main effects (lifestyle variables used to construct the strata) were added to the fixed part of model 1.

### Model 3: Main effects model with confounders

Model 3 is an expansion of model 2 by the addition of confounders (age and TDI were centred at their means for meaningful interpretation of the intercept).

For each model, the variance partition coefficient (VPC) was calculated as the proportion of the total variance attributable to between strata variance and expressed in percentage:

$${VPC}=\frac{{Variance\; between\; strata}}{{Variance\; between\; strata}+{Variance\; within\; strata}}\,\times 100$$

The VPC in logistic models is computed differently than in general linear models as the individual-level variance is not estimated. The ${\sigma }_{e}^{2}$ is therefore set at the variance of the standard logistic distribution or $\frac{{\pi }^{2}}{3}=3.29,$ where π signifies the constant 3.142. We expected a reduction in the VPC in models 2 and 3 compared to model 1 as some between-stratum variance would be explained by the inclusion of predictor variables.

We also calculated the proportional change in variance (PCV) as a measure of the amount of between stratum variance due to additive effects, expressed as a percentage.

$${PCV}=\,\frac{{\sigma }_{\mu 2}^{2}-\,{\sigma }_{\mu 1}^{2}}{{\sigma }_{\mu 1}^{2}}\,\times 100$$

where ${\sigma }_{\mu 1}^{2}$ is the between stratum variance in model 1 and ${\sigma }_{\mu 2}^{2}$ is the between stratum variance in models 2 or 3.

We also calculated the predicted BMI and obesity risk for each individual strata and ranked them in ascending order. We also identified strata where predicted BMI or obesity odds were influenced by interactive effects, both positive and negative. This was achieved by isolating each stratum’s residuals and comparing the 95% confidence intervals for estimates with and without the random effect. No overlap of the intervals in a stratum was indicative of the presence of a multiplicative effect for that stratum.

All procedures were performed in Stata 17 (StataCorp LP, College Station, TX, USA). The command runmlwin was used for the multilevel models [43].

## Results

### Participant characteristics

As shown in Table 1, median BMI values were in the overweight range, and 23% of males and 17% of females had obesity. While most men and women met the recommendations for physical activity and sleep duration, few met those for fruit and vegetable and alcohol intake.

**Table 1:** Descriptive statistics.

		Males *n* = 139,540	Females *n* = 125,455
Lifestyle variables			
Smoking			
Current	*N* (%)	15,619 (11.2)	10,129 (8.0)
Previous	*N* (%)	56,536 (40.5)	44,480 (35.5)
Never	*N* (%)	67,385 (48.3)	70,846 (56.5)
Fruit and vegetable			
Not meets	*N* (%)	101,619 (72.8)	77,109 (61.5)
Meets	*N* (%)	37,921 (27.2)	48,346 (38.5)
Physical activity			
Not meets	*N* (%)	24,943 (17.9)	21,118 (16.8)
Meets	*N* (%)	114,597 (82.1)	104,337 (83.2)
Alcohol			
Not meets	*N* (%)	104,785 (75.1)	65,334 (52.1)
Meets	*N* (%)	34,755 (24.9)	60,121 (47.9)
Sleep			
Not meets	*N* (%)	34,145 (24.5)	28,721 (22.9)
Meets	*N* (%)	105,395 (75.5)	96,734 (77.1)
Confounders			
Age	Mean (SD)	56.7 (8.0)	55.7 (7.9)
Townsend Deprivation Index	Mean (SD)	−1.6 (2.9)	−1.7 (2.7)
Ethnicity			
White	*N* (%)	130,132 (93.3)	115,878 (92.4)
Non-White	*N* (%)	9408 (6.7)	9577 (7.6)
Employment			
Employed	*N* (%)	87,975 (63.1)	75,454 (60.1)
Not currently employed or retired	*N* (%)	51,565 (36.9)	50,001 (39.9)
Outcome			
BMI	Median (IQR)	27.1 (24.9–29.7)	25.4 (23.1–28.5)
BMI status			
<18.5 (Underweight)	*N* (%)	258 (0.2)	891 (0.7)
18.5–24.9 (Normal weight)	*N* (%)	35,746 (25.6)	56,454 (45.0)
25–29.9 (Overweight)	*N* (%)	71,193 (51.0)	46,439 (37.0)
>30 (Obese)	*N* (%)	32,343 (23.2)	21,671 (17.3)

### General linear MAIHDA models for BMI

Pairwise correlations between the lifestyle behaviours and confounders were generally low ( < 0.2), and the VIFs for all predictors were below 1.6 for both males and females, indicating that multicollinearity was unlikely to bias the estimates.

Results for the general linear MAIHDA models for BMI are given in Table 2. In model 1, the level 2 (between-stratum) variance was 0.7 for males and females, while the level 1 (between-individual, within-strata) variance was 15% for males and 20% for females. The VPC was 4% for both sexes, indicating that only 4% of the variances in BMI were attributable to differences between strata as opposed to differences between individuals, within strata.

**Table 2:** MAIHDA models for BMI.

	Males			Females		
	Null Model	Main effects model	Main effects model plus confounders^a^	Null Model	Main effects Model	Main effects Model plus confounders^a^
	β (95% CI)	β (95% CI)	β (95% CI)	β (95% CI)	β (95% CI)	β (95% CI)
Intercept	28.04 (27.80, 28.29)	29.64 (29.45, 29.84)	29.82 (29.60, 30.05)	26.87 (26.61, 27.12)	28.36 (28.18, 28.53)	28.60 (28.39, 28.81)
Smoking						
Previous		–	–		–	–
Current		−0.90 (-1.10, -0.71)	−0.93 (−1.13, −0.74)		−0.52 (−0.69, −0.34)	−0.50 (−0.67, −0.33)
Never		−1.03 (−1.20, −0.86)	−0.99 (−1.17, −0.82)		−0.54 (−0.69, −0.40)	−0.44 (−0.58, −0.30)
Fruit & vegetable						
Not meets		–	–		–	–
Meets		0.11 (−0.04, 0.26)	0.10 (−0.04, 0.25)		−0.32 (−0.45, −0.20)	−0.39 (−0.51, −0.26)
Physical activity						
Not meets		–	–		–	–
Meets		−1.07 (−1.22, −0.92)	−1.08 (−1.23, −0.93)		−1.46 (−1.59, −1.33)	−1.50 (−1.63, −1.37)
Alcohol						
Not meets		–	–		–	–
Meets		−0.32 (−0.47, −0.17)	−0.31 (−0.47, −0.16)		0.19 (0.06, 0.32)	0.17 (0.04, 0.29)
Sleep						
Not meets		–	–		–	
Meets		−0.66 (−0.81, −0.51)	−0.64 (−0.79, −0.49)		−0.63 (−0.76, −0.50)	−0.56 (−0.69, −0.43)
Level 2 variance	0.70 (0.40, 0.99)	0.05 (0.02, 0.07)	0.05 (0.02, 0.07)	0.76 (0.44, 1.09)	0.03 (0.01, 0.04)	0.02 (0.01, 0.04)
Level 1 variance	15.15 (15.04, 15.26)	15.15 (15.04, 15.26)	15.13 (15.02, 15.24)	19.66 (19.51, 19.82)	19.66 (19.51, 19.82)	19.47 (19.32, 19.62)
VPC	4.43%	0.33%	0.33%	3.76%	0.15%	0.15%
PCV^b^		92.80%	92.76%		96.01%	96.17%

^a^Confounders were Age, ethnicity, tdi and employment status.

^b^All estimated PCVs are relative to the null model.

After adding the main effects for predictor variables in model 2, the VPCs reduced to 0.3% for males and 0.2% for females. The small VPCs indicated that most of the between-strata variance was explained by additive effects, with minimal contribution from multiplicative effects. This was further supported by the model 2 PCV estimates, which showed that 93% of the between-strata variance for males and 96% for females were explained by additive main effects, highlighting the minimal role of interactive effects. On adjusting for the confounders in model 3, VPCs and PCVs remained similar.

#### Predictions from the linear models

Table 3 shows the five highest and lowest strata ranked by their predicted mean BMI from model 3. The full list of strata ranked by their predicted mean BMI is given in Supplementary Tables 2 and 3. For both sexes, most of the lifestyle recommendations were met by the strata that had the lowest predicted mean BMI. For instance, among males, the strata of never smokers who met all four lifestyle behaviour recommendations had the lowest predicted mean BMI of 26.7 kg/m^2^. Conversely, the strata that did not meet most of the recommendations were among the highest predicted mean BMI. For example, the strata with males who were previous smokers and did not meet any of the lifestyle behaviour recommendations except for daily fruit and vegetable intake, had the highest predicted mean BMI of 30.0 kg/m^2^. Additionally, adherence to the physical activity and sleep guidelines was observed in all five strata with the lowest predicted mean BMI for both males and females.

**Table 3:** The five strata with the lowest and highest BMI.

Strata ID	*N*	Fruit & veg	Physical activity	Sleep	Alcohol	Smoking	Observed BMI	Predicted BMI including random intercept (95% CI)	Predicted BMI excluding random intercept (95% CI)	Random intercept (95% CI)
Females: strata with the lowest predicted BMI										
11101	1043	Meets	Meets	Meets	Not meets	Current	25.5	25.7 (25.4, 26.0)	25.6 (25.4, 25.8)	0.09 (−0.14, 0.33)
11102	8055	Meets	Meets	Meets	Not meets	Never	25.5	25.8 (25.5, 26.0)	25.6 (25.5, 25.8)	0.10 (−0.06, 0.27)
01101	2847	Not meets	Meets	Meets	Not meets	Current	25.5	25.8 (25.5, 26.1)	26.0 (25.8, 26.1)	−0.17 (−0.37, 0.02)
11112	10660	Meets	Meets	Meets	Meets	Never	25.6	25.9 (25.6, 26.1)	25.8 (25.6, 26.0)	0.03 (−0.12, 0.20)
11111	590	Meets	Meets	Meets	Meets	Current	26.1	26.0 (25.6, 26.3)	25.8 (25.6, 25.9)	0.20 (−0.05, 0.46)
Females: strata with the highest predicted BMI										
00011	176	Not meets	Not meets	Not meets	Meets	Current	28.2	28.2 (27.9, 28.6)	28.2 (28.0, 28.4)	0.001 (−0.29, 0.31)
00002	871	Not meets	Not meets	Not meets	Not meets	Never	28.3	28.4 (28.1, 28.7)	28.1 (27.9, 28.3)	0.27 (0.038, 0.51)
10010	284	Meets	Not meets	Not meets	Meets	Previous	28.4	28.4 (28.1, 28.7)	28.3 (28.1, 28.5)	0.07 (−0.21, 0.35)
00000	727	Not meets	Not meets	Not meets	Not meets	Previous	28.6	28.7 (28.4, 29.0)	28.6 (28.4, 28.7)	0.10 (−0.14, 0.35)
00010	518	Not meets	Not meets	Not meets	Meets	Previous	29.1	29.0 (28.6, 29.3)	28.7 (28.5, 28.9)	0.23 (−0.03, 0.49)
Males: strata with the lowest predicted BMI										
11112	4170	Meets	Meets	Meets	Meets	Never	26.5	26.7 (26.4, 26.9)	26.8 (26.6, 27.0)	−0.18 (−0.39, 0.02)
01112	8952	Not meets	Meets	Meets	Meets	Never	26.6	26.7 (26.5, 27.0)	26.7 (26.5, 26.9)	−0.002 (−0.20, 0.19)
01101	6179	Not meets	Meets	Meets	Not meets	Current	27.0	27.0 (26.7, 27.3)	27.1 (26.9, 27.3)	−0.09 (−0.30, 0.11)
01111	997	Not meets	Meets	Meets	Meets	Current	27.0	27.0 (26.7, 27.4)	26.8 (26.6, 27.0)	0.24 (−0.02, 0.50)
11102	8451	Meets	Meets	Meets	Not meets	Never	27.0	27.1 (26.8, 27.4)	27.2 (27.0, 27.4)	−0.05 (−0.25, 0.14)
Males: strata with the highest predicted BMI										
10100	1139	Meets	Not meets	Meets	Not meets	Previous	29.4	29.4 (29.1, 29.8)	29.2 (29.0, 29.4)	0.19 (−0.05, 0.45)
10010	147	Meets	Not meets	Not meets	Meets	Previous	29.4	29.5 (29.1, 30.0)	29.6 (29.3, 29.8)	−0.01 (−0.38, 0.35)
00010	445	Not meets	Not meets	Not meets	Meets	Previous	29.7	29.7 (29.3, 30.1)	29.5 (29.2, 29.7)	0.24 (−0.06, 0.54)
00000	1642	Not meets	Not meets	Not meets	Not meets	Previous	29.7	29.9 (29.6, 30.2)	29.8 (29.6, 30.0)	0.11 (−0.12, 0.35)
10000	430	Meets	Not meets	Not meets	Not meets	Previous	30.0	30.0 (29.6, 30.4)	29.9 (29.7, 30.1)	0.12 (−0.18, 0.43)

All estimates are from the main effects plus confounders model.

While there was little to no evidence of multiplicative effects across most strata, interactive effects were observed for one specific combination of lifestyle behaviours only – male current smokers meeting only the physical activity recommendations. Non-overlap of the 95% CIs for predicted BMI when including (26.9–27.5 kg/m^2^) vs. excluding (27.5–28.0 kg/m^2^) the random intercept in the stratum indicated the presence of a multiplicative effect.

### Binary logistic MAIHDA models

The results of the binary logistic models for obesity vs. normal weight is given in Table 4. The VPCs from model 1 indicated that 6% of the total variance for females and 7% for males were explained by differences between strata as opposed to between individuals, within strata. These reduced to near zero values (0.004% for males and 0.010% for females) in model 2 on addition of the fixed effects. The model 2 PCVs of 98% for males and 93% for females indicated that most of the between-strata variances were explained by additive main effects compared to multiplicative effects. VPCs and PCVs remained largely unchanged in model 3 on further adjustment for the confounders, indicating only a very minor influence of the confounding variables.

**Table 4:** MAIHDA models for obesity status.

	Females			Males		
	Null model	Main effects model	Main effects model+	Null model	Main effects model	Main effects model+
	OR (95% CI)	OR (95% CI)	OR (95% CI)	OR (95% CI)	OR (95% CI)	OR (95% CI)
Intercept	0.51 (0.44, 0.58)	1.12 (1.03, 1.21)	1.26 (1.12, 1.42)	1.11 (0.96, 1.28)	3.13 (2.78, 3.54)	3.90 (3.37, 4.52)
Smoking						
Previous		–	–		–	–
Current		0.77 (0.71, 0.84)	0.76 (0.70, 0.83)		0.53 (0.47, 0.60)	0.53 (0.47, 0.60)
Never		0.76 (0.72, 0.81)	0.81 (0.76, 0.86)		0.50 (0.45, 0.55)	0.51 (0.46, 0.57)
Fruit & vegetable						
Not meets		–	–		–	–
Meets		0.80 (0.76, 0.85)	0.77 (0.73, 0.82)		1.02 (0.92, 1.12)	1.01 (0.92, 1.11)
Physical activity						
Not meets		–	–		–	–
Meets		0.47 (0.44, 0.50)	0.45 (0.43, 0.48)		0.53 (0.48, 0.59)	0.53 (0.48, 0.58)
Alcohol						
Not meets		–	–		–	–
Meets		1.09 (1.02, 1.15)	1.08 (1.02, 1.14)		0.77 (0.70, 0.84)	0.77 (0.70, 0.84)
Sleep						
Not meets		–	–		–	–
Meets		0.71 (0.67, 0.75)	0.74 (0.69, 0.78)		0.69 (0.63, 0.76)	0.70 (0.63, 0.76)
Level 2 variance	0.19 (0.11, 0.27)	0.004 (0.0008, 0.007)	0.004 (0.0006, 0.007)	0.25 (0.14, 0.36)	0.01 (0.007, 0.02)	0.01 (0.007, 0.02)
Level 1 variance	–	–	–	–	–	–
VPC	5.63%	0.13%	0.12%	7.12%	0.52%	0.52%
PCV	–	97.77%	97.93%	–	93.08%	93.15%

^a^Confounders were Age, ethnicity, tdi and employment status.

^b^All estimated PCVs are relative to the null model.

#### Predictions from the logistic models

Table 5 shows the top five and bottom five strata ranked by their predicted obesity probabilities from Model 3. For the strata with the lowest predicted probability of obesity, most lifestyle recommendations were followed, and this trend was consistent for both males and females. For example, among males, the strata of never smokers who adhered to all four lifestyle recommendations had the lowest predicted obesity probability (33%). Conversely, the strata that did not follow most of the lifestyle recommendations had the highest predicted probability of obesity. For instance, among males, the strata of previous smokers who did not meet any of the lifestyle recommendations had the highest predicted obesity probability estimates (79%). The full list of strata ranked by their predicted obesity probabilities are given in Supplementary Tables 4 and 5. Furthermore, as observed with BMI, all five strata with the lowest predicted probability of obesity for both sexes consistently followed the physical activity and sleep guidelines. We also observed that the same strata of male current smokers meeting only the physical activity recommendations displayed a non-overlap of 95% CIs (0.40–0.49, 0.49–0.56), indicating the presence of interactive effects on predicted obesity probability.

**Table 5:** The five strata with the lowest and highest predicted probability of obesity.

Strata ID	*N*	Fruit & veg	Physical activity	Sleep	Alcohol	Smoking	Observed BMI	Predicted probability of obesity including random intercept (95% CI)	Predicted probability of obesity excluding random intercept (95% CI)	Random intercept (95% CI)
Females: strata with the lowest predicted probability of obesity										
11101	1043	Meets	Meets	Meets	Not meets	Current	0.18	0.20 (0.18, 0.22)	0.20 (0.18, 0.21)	−0.001 (−0.10, 0.10)
11102	8055	Meets	Meets	Meets	Not meets	Never	0.19	0.21 (0.19, 0.23)	0.21 (0.20, 0.22)	0.004 (−0.07, 0.08)
11111	590	Meets	Meets	Meets	Meets	Current	0.23	0.22 (0.19, 0.25)	0.21 (0.20, 0.23)	0.03 (−0.07, 0.14)
11112	10660	Meets	Meets	Meets	Meets	Never	0.20	0.22 (0.21, 0.24)	0.22 (0.21, 0.24)	0.001 (−0.07, 0.07)
01101	2847	Not meets	Meets	Meets	Not meets	Current	0.20	0.23 (0.21, 0.25)	0.24 (0.23, 0.26)	−0.06 (−0.16, 0.02)
Females: strata with the highest predicted probability of obesity										
10010	284	Meets	Not meets	Not meets	Meets	Previous	0.48	0.51 (0.47, 0.55)	0.51 (0.49, 0.53)	−0.005 (−0.12, 0.11)
00002	871	Not meets	Not meets	Not meets	Not meets	Never	0.48	0.51 (0.48, 0.55)	0.50 (0.48, 0.52)	0.038 (−0.06, 0.14)
00012	1053	Not meets	Not meets	Not meets	Meets	Never	0.46	0.52 (0.48, 0.55)	0.52 (0.50, 0.55)	−0.03 (−0.13, 0.06)
00000	727	Not meets	Not meets	Not meets	Not meets	Previous	0.53	0.56 (0.52, 0.59)	0.55 (0.53, 0.57)	0.005 (−0.10, 0.11)
00010	518	Not meets	Not meets	Not meets	Meets	Previous	0.55	0.58 (0.54, 0.61)	0.57 (0.55, 0.59)	0.007 (−0.10, 0.11)
Males: strata with the lowest predicted probability of obesity										
11112	4170	Meets	Meets	Meets	Meets	Never	0.28	0.33 (0.29, 0.37)	0.37 (0.34, 0.39)	−0.15 (−0.29, −0.01)
01112	8952	Not meets	Meets	Meets	Meets	Never	0.29	0.34 (0.31, 0.38)	0.36 (0.34, 0.39)	−0.08 (−0.21, 0.03)
11111	275	Meets	Meets	Meets	Meets	Current	0.40	0.40 (0.34, 0.47)	0.38 (0.34, 0.41)	0.11 (−0.09, 0.32)
01111	997	Not meets	Meets	Meets	Meets	Current	0.37	0.41 (0.36, 0.46)	0.37 (0.34, 0.40)	0.14 (−0.03, 0.31)
01101	6179	Not meets	Meets	Meets	Not meets	Current	0.35	0.41 (0.37, 0.45)	0.43 (0.41, 0.47)	−0.10 (−0.24, 0.02)
Males: strata with the highest predicted probability of obesity										
10010	147	Meets	Not meets	Not meets	Meets	Previous	0.67	0.74 (0.68, 0.79)	0.75 (0.72, 0.78)	−0.04 (−0.27, 0.18)
10100	1139	Meets	Not meets	Meets	Not meets	Previous	0.73	0.76 (0.72, 0.79)	0.73 (0.71, 0.75)	0.13 (−0.03, 0.30)
00010	445	Not meets	Not meets	Not meets	Meets	Previous	0.74	0.76 (0.72, 0.80)	0.75 (0.72, 0.77)	0.09 (−0.10, 0.29)
10000	430	Meets	Not meets	Not meets	Not meets	Previous	0.74	0.79 (0.75, 0.82)	0.79 (0.77, 0.81)	−0.03 (−0.23, 0.16)
00000	1642	Not meets	Not meets	Not meets	Not meets	Previous	0.76	0.79 (0.76, 0.82)	0.79 (0.77, 0.81)	0.01 (−0.14, 0.17)

All estimates are from the main effects plus confounders model.

Given that the manuscript focuses on obesity as the primary clinical outcome, we have presented only the main results for the linear BMI models and the logistic models comparing obesity with normal weight. Logistic models comparing overweight with normal weight are reported separately in Supplementary Tables 6–8.

## Discussion

Lifestyle behaviours such as physical activity, diet, smoking, alcohol intake, and sleep influence a range of health outcomes including obesity [4, 5, 12–15]. While prior studies have explored associations between individual behaviours and obesity risk, this study advances knowledge by investigating their combined relationship with BMI and obesity risk [13–15, 27, 28]. This is important as these modifiable lifestyle behaviours do not occur in isolation and may interact. Using MAIHDA, our key findings were that 1) most variation in BMI and obesity risk was due to differences between individuals (within the same strata) rather than between strata, 2) the influence of lifestyle behaviours on BMI and obesity risk was largely additive, with minimal multiplicative effects, 3) practically, this manifested as the strata meeting more number of lifestyle recommendations had lower predicted BMI and obesity risk, and 4) meeting the physical activity and sleep guidelines appeared most consistently in the strata with the lowest predicted BMI and obesity risk.

To our knowledge, this is the first study to apply MAIHDA to behavioural obesity epidemiology to examine the combined and interactive associations of multiple lifestyle behaviours with obesity risk. As highlighted by many studies, obesity often has multifactorial lifestyle risk factors [44–47]. Therefore, MAIHDA is particularly relevant in the context of obesity epidemiology, as it allows the investigation of multiple behaviours and their interactions. The present study found that most of the total variation in BMI and probability of obesity was explained by between-individual, within-strata differences as opposed to between-strata differences, as shown by VPC estimates of 4% for males and 7% for females. For reference, the VPCs of MAIHDA null models are typically around 5%, rarely exceeding 10% [34, 45, 48]. This demonstrates the consistency of the MAIHDA approach across different contexts. Similar patterns have been observed in previous MAIHDA research reporting comparable variance parameters (VPC ~ 5%, PCV ~ 95%) [48–53]. The primarily within-strata effects suggest that while combined healthy lifestyle behaviours play an important role in lowering BMI and obesity risk, most variance in mean BMI and obesity risk is explained by other factors not examined here (e.g. neighbourhood, access to parks, job nature, and wider dietary intake).

Our findings showed that the effects of meeting vs. not meeting lifestyle behaviour recommendations on BMI and obesity risk were predominantly additive rather than multiplicative. This means that the influence of meeting or not meeting each lifestyle factor on obesity risk was largely independent and added to one another, rather than being dependent on each other and their combined effect being the product of their individual effects. This was demonstrated by the model 2 and 3 PCVs and the substantial reduction in VPCs after adding the main effects. This finding aligns with previous research in which an unhealthy diet and physical inactivity combined additively increased several cardio-metabolic outcomes such as Type 2 Diabetes, dyslipidemia, and hypertension [54, 55]. Similarly, Layman et al., highlighted the additive effect of physical activity and diet on improving body composition and lowering of body fat [56]. These findings suggest additional benefits from following more individual lifestyle recommendations, rather than synergistic effects between their combinations. In other words, meeting each additional lifestyle behaviour recommendation is associated with lower BMI and obesity risk. For example, even if three out of the five guidelines are met, meeting one or both of the remaining guidelines may further decrease risk.

Predictions from the final models indicated that strata meeting most recommendations had lower predicted BMI and obesity risk, while those not meeting most had higher predicted BMI and obesity risk. For instance, males who never smoked and met all recommendations, had the lowest predicted mean BMI (26.7 kg/m^2^) and probability of obesity (33%). In comparison, the predicted BMI and obesity probability were 4.5 kg/m^2^ and 46%, respectively, higher in males who were previous smokers and met none of the lifestyle behaviour recommendations. These differences in the highest and lowest ranked strata underscore the importance of meeting all the lifestyle recommendations in reducing BMI and obesity risk. This aligns with previous research emphasizing benefits of multiple healthy lifestyle behaviours in reducing obesity [18, 47, 53].

It is important to align the core message with the primary study aim, which focuses on the combined effect of multiple lifestyle behaviours rather than the individual impact of any single behaviour. While certain behaviours, such as physical activity, sleep, and smoking appear to contribute more to BMI and obesity outcomes in our study, these must be interpreted in the context of the combination with other behaviours and not in isolation.

The study findings have implications for future research aimed at mitigating obesity risk. By using MAIHDA to explore obesity risk differences between lifestyle behaviour combinations, we have provided evidence of additive benefits of meeting recommendations. While this means that meeting each of these individual lifestyle behaviour recommendations is important, and some may be relatively more influential, at the population level, emphasis should be on meeting as many as possible to maximise the weight-related benefits of these recommendations. Likewise, at the individual level, adopting each additional behaviour confers additive benefits. However, our study also highlighted the greater influence of factors other than the lifestyle behaviour combinations, which could include aspects such as the built environment, neighbourhood, and access to public walkways. We therefore recommend that future research include such structural factors to better understand their combined impact on obesity risk and related conditions using primary data and other large-scale datasets. Furthermore, future work could also model lifestyle behaviours as continuous exposures or as part of an aggregate lifestyle score, enabling the assessment of potential dose-response and/or non-linear relationships alongside guideline-based thresholds.

### Strengths and limitations

The study has several strengths. First, the use of MAIHDA enabled a more detailed exploration of the stratum-specific intersectional effects of the lifestyle behaviours, providing an advantage over traditional methods, which are often limited in the number of interactions they can investigate [36, 38, 43, 51, 52]. While other approaches such as LCA categorize individuals into distinct subgroups based on patterns of behaviour, they may fail to capture the complex relationships between behaviours and outcomes at the individual level. In contrast, MAIHDA allows for a more dynamic and flexible modelling approach, providing more precise estimates, especially when dealing with smaller groups. Another strength is the large sample size, which was crucial in enabling us to demonstrate the application of MAIHDA to explore variation in BMI and obesity risk according to different lifestyle behaviour combinations. Lastly, the use of nationally recommended guidelines for these lifestyle behaviours enhances the generalizability of our findings to the broader population.

However, there are several limitations to consider. First, while we focused on the key lifestyle behaviours influencing obesity risk, other behavioural factors that also could impact obesity were not included in the analysis. Second, certain covariates, such as educational attainment and income, were not adjusted for due to high levels of non-response (i.e. missing data), and their potential confounding effects on the relationship between lifestyle behaviours and obesity risk should be considered in future research. Third, since the analysis relied on self-reported data for the lifestyle behaviours, recall bias might have influenced the results. Fourth, although each of these lifestyle factors have been shown to causally impact body weight, there is also evidence that greater adiposity itself may lead to reductions in physical activity and increase in sedentary behaviour, indicating possible reverse causality [57, 58]. Given the study’s cross-sectional design, the analysis cannot establish temporality or address reverse causality. Finally, the UK Biobank sample is not fully representative of the broader UK population, which could introduce bias and limit the applicability of our findings to the general population.

## Conclusions

Our findings highlight substantial differences in mean BMI and obesity risk between groups of adults who meet different combinations of lifestyle behaviour guidelines. However, most of this variation in the outcomes was attributed to differences between individuals within strata, as opposed to differences between strata. Overall, we found predominantly additive effects and minimal evidence of interactive effects of the lifestyle behaviours. This has important public health implications highlighting that there are additive benefits of meeting a greater number of public health lifestyle behaviour recommendations in the context of reducing BMI and obesity risk.

## Supplementary information

Supplementary materials

## Data Availability

Data from the UK Biobank can be accessed by researchers for health-related studies conducted in the public interest. Further details are available at https://www.ukbiobank.ac.uk/use-our-data/.

## Code availability

Available from the first author upon request.

## References

[1] NHS. Overweight and obesity in adults - NHS England Digital. 2022. https://digital.nhs.uk/data-and-information/publications/statistical/health-survey-for-england/2021/overweight-and-obesity-in-adults.

[2] Janssen F, Bardoutsos A, Vidra N. Obesity prevalence in the long-term future in 18 European Countries and in the USA. Obes Facts. 2020;13:514–27. 10.1159/000511023. 33075798 10.1159/000511023 PMC7670332

[3] GOV.UK. Government plans to tackle obesity in England. Department of Health and Social Care Media Centre. 2023. https://healthmedia.blog.gov.uk/2023/06/07/government-plans-to-tackle-obesity-in-england/.

[4] WHO. Obesity and overweight. 2024. https://www.who.int/news-room/fact-sheets/detail/obesity-and-overweight.

[5] Masood B, Moorthy M. Causes of obesity: a review. Clin Med. 2023;23:284–91. 10.7861/clinmed.2023-0168. 10.7861/clinmed.2023-0168 PMC10541056 37524429

[6] Gray CL, Messer LC, Rappazzo KM, Jagai JS, Grabich SC, Lobdell DT. The association between physical inactivity and obesity is modified by five domains of environmental quality in U.S. adults: a cross-sectional study. PLoS One. 2018;13:e0203301 10.1371/journal.pone.0203301. 30161196 10.1371/journal.pone.0203301 PMC6117021

[7] Silveira EA, Mendonça CR, Delpino FM, Elias Souza GV, Pereira de Souza Rosa L, de Oliveira C, et al. Sedentary behavior, physical inactivity, abdominal obesity and obesity in adults and older adults: a systematic review and meta-analysis. Clin Nutr ESPEN. 2022;50:63–73. 10.1016/j.clnesp.2022.06.001. 35871953 10.1016/j.clnesp.2022.06.001

[8] NHS. Obesity. NHS choices. 2023. https://www.nhs.uk/conditions/obesity/causes/.

[9] Ahmadi MN, Inan-Eroglu E, Mishra GD, Salis A, Stamatakis E. Associations of changes in physical activity and diet with incident obesity and changes in adiposity: longitudinal findings from the UK Biobank. Prev Med. 2023;168:107435 10.1016/j.ypmed.2023.107435. 36746246 10.1016/j.ypmed.2023.107435

[10] Paudel S, Del Pozo Cruz B, Inan-Eroglu E, Ahmadi M, Stamatakis E. Associations of changes in physical activity and discretionary screen time with incident obesity and adiposity changes: longitudinal findings from the UK Biobank. Int J Obes. 2022;46:597–604. 10.1038/s41366-021-01033-8. 10.1038/s41366-021-01033-8 34853431

[11] Sanchez-Lastra MA, Ding D, Del Pozo Cruz B, Dalene KE, Ayán C, Ekelund U, et al. Joint associations of device-measured physical activity and abdominal obesity with incident cardiovascular disease: a prospective cohort study. Br J Sports Med. 2024;58:196–203. 10.1136/bjsports-2023-107252. 37940366 10.1136/bjsports-2023-107252 PMC10894840

[12] Nour M, Lutze SA, Grech A, Allman-Farinelli M. The relationship between vegetable intake and weight outcomes: a systematic review of cohort studies. Nutrients. 2018;10:1626 10.3390/nu10111626. 30400139 10.3390/nu10111626 PMC6266069

[13] Heath L, Jebb SA, Aveyard P, Piernas C. Obesity, metabolic risk and adherence to healthy lifestyle behaviours: prospective cohort study in the UK Biobank. BMC Med. 2022;20:65. 10.1186/s12916-022-02236-0. 35164754 10.1186/s12916-022-02236-0 PMC8845299

[14] Antza C, Kostopoulos G, Mostafa S, Nirantharakumar K, Tahrani A. The links between sleep duration, obesity and type 2 diabetes mellitus. J Endocrinol. 2021;252:125–41. 10.1530/JOE-21-0155. 34779405 10.1530/JOE-21-0155 PMC8679843

[15] Rassy N, Van Straaten A, Carette C, Hamer M, Rives-Lange C, Czernichow S. Association of healthy lifestyle factors and obesity-related diseases in adults in the UK. JAMA Netw Open. 2023;6:e2314741 10.1001/jamanetworkopen.2023.14741. 37234008 10.1001/jamanetworkopen.2023.14741 PMC10220514

[16] Brady EM, Bodicoat DH, Hall AP, Khunti K, Yates T, Edwardson C, et al. Sleep duration, obesity and insulin resistance in a multi-ethnic UK population at high risk of diabetes. Diabetes Res Clin Pract. 2018;139:195–202. 10.1016/j.diabres.2018.03.010. 29526681 10.1016/j.diabres.2018.03.010

[17] Bacaro V, Ballesio A, Cerolini S, Vacca M, Poggiogalle E, Donini LM, et al. Sleep duration and obesity in adulthood: an updated systematic review and meta-analysis. Obes Res Clin Pract. 2020;14:301–9. 10.1016/j.orcp.2020.03.004. 32527625 10.1016/j.orcp.2020.03.004

[18] Chudasama YV, Khunti K, Gillies CL, Dhalwani NN, Davies MJ, Yates T, et al. Healthy lifestyle and life expectancy in people with multimorbidity in the UK Biobank: a longitudinal cohort study. PLoS Med. 2020;17:e1003332 10.1371/journal.pmed.1003332. 32960883 10.1371/journal.pmed.1003332 PMC7508366

[19] Wu Y, Zhai L, Zhang D. Sleep duration and obesity among adults: a meta-analysis of prospective studies. Sleep Med. 2014;15:1456–62. 10.1016/j.sleep.2014.07.018. 25450058 10.1016/j.sleep.2014.07.018

[20] Li Q. The association between sleep duration and excess body weight of the American adult population: a cross-sectional study of the national health and nutrition examination survey 2015-6. BMC Public Health. 2021;21:335. 10.1186/s12889-021-10369-9. 33573618 10.1186/s12889-021-10369-9 PMC7879643

[21] O’Donovan G, Inan-Eroglu E, Stamatakis E, Hamer M. Alcohol drinking in one’s thirties and forties is associated with body mass index in men, but not in women: a longitudinal analysis of the 1970 British Cohort Study. Prev Med. 2021;153:106811. 10.1016/j.ypmed.2021.106811. 34560097 10.1016/j.ypmed.2021.106811

[22] Sneve M, Jorde R. Cross-sectional study on the relationship between body mass index and smoking, and longitudinal changes in body mass index in relation to change in smoking status: the Tromso Study. Scand J Public Health. 2008;36:397–407. 10.1177/1403494807088453. 18539694 10.1177/1403494807088453

[23] Munafò MR, Tilling K, Ben-Shlomo Y. Smoking status and body mass index: a longitudinal study. Nicotine Tob Res. 2009;11:765–71. 10.1093/ntr/ntp062. 19443785 10.1093/ntr/ntp062

[24] Mackay DF, Gray L, Pell JP. Impact of smoking and smoking cessation on overweight and obesity: Scotland-wide, cross-sectional study on 40,036 participants. BMC Public Health. 2013;13:348. 10.1186/1471-2458-13-348. 23587253 10.1186/1471-2458-13-348 PMC3636072

[25] Dare S, Mackay DF, Pell JP. Relationship between smoking and obesity: a cross-sectional study of 499,504 middle-aged adults in the UK general population. PLoS One. 2015;10:e0123579 10.1371/journal.pone.0123579. 25886648 10.1371/journal.pone.0123579 PMC4401671

[26] Pedisic Zeljko. Measurement issues and poor adjustments for physical activity and sleep undermine sedentary behaviour research—The focus should shift to the balance between sleep, sedentary behaviour, standing and activity. Kinesiology. 2014;46:135–46.

[27] Gennuso KP, Thraen-Borowski KM, Schlam TR, LaRowe TL, Fiore MC, Baker TB, et al. Smokers’ physical activity and weight gain one year after a successful versus unsuccessful quit attempt. Prev Med. 2014;67:189–92. 10.1016/j.ypmed.2014.07.040. 25091879 10.1016/j.ypmed.2014.07.040 PMC4457287

[28] Saunders TJ, Gray CE, Poitras VJ, Chaput JP, Janssen I, Katzmarzyk PT, et al. Combinations of physical activity, sedentary behaviour and sleep: relationships with health indicators in school-aged children and youth. Appl Physiol Nutr Metab. 2016;41:S283–93. 10.1139/apnm-2015-0626. 27306434 10.1139/apnm-2015-0626

[29] Kaufman A, Augustson EM, Patrick H. Unraveling the relationship between smoking and weight: the role of sedentary behavior. J Obes. 2012;2012:735465. 10.1155/2012/735465. 21961058 10.1155/2012/735465 PMC3180774

[30] Nurwanti E, Uddin M, Chang JS, Hadi H, Syed-Abdul S, Su EC, et al. Roles of sedentary behaviors and unhealthy foods in increasing the obesity risk in adult men and women: a cross-sectional national study. Nutrients. 2018;10:704 10.3390/nu10060704. 29857537 10.3390/nu10060704 PMC6024814

[31] Kim SY, Kim HJ. Obesity risk was associated with alcohol intake and sleep duration among Korean men: the 2016-2020 Korea National Health and Nutrition Examination Survey. Nutrients. 2024;16:3950 10.3390/nu16223950. 39599735 10.3390/nu16223950 PMC11597622

[32] Leech RM, McNaughton SA, Timperio A. The clustering of diet, physical activity and sedentary behavior in children and adolescents: a review. Int J Behav Nutr Phys Act. 2014;11:4. 10.1186/1479-5868-11-4. 24450617 10.1186/1479-5868-11-4 PMC3904164

[33] Verswijveren SJ, Dingle S, Donnelly AE, et al. How are different clusters of physical activity, sedentary, sleep, smoking, alcohol, and dietary behaviors associated with cardiometabolic health in older adults? A cross-sectional latent class analysis. JASSB. 2023;2:16 10.1186/s44167-023-00025-5. 40217447 10.1186/s44167-023-00025-5 PMC11960331

[34] Niu M, Chen J, Hou R, Sun Y, Xiao Q, Pan X, et al. Emerging healthy lifestyle factors and all-cause mortality among people with metabolic syndrome and metabolic syndrome-like characteristics in NHANES. J Transl Med. 2023;21:239. 10.1186/s12967-023-04062-1. 37005663 10.1186/s12967-023-04062-1 PMC10068159

[35] Liberali R, Del Castanhel F, Kupek E, Assis MAA. Latent class analysis of lifestyle risk factors and association with overweight and/or obesity in children and adolescents: systematic review. Child Obes. 2021;17:2–15. 10.1089/chi.2020.0115. 33306451 10.1089/chi.2020.0115

[36] Merlo J. Multilevel analysis of individual heterogeneity and discriminatory accuracy (MAIHDA) within an intersectional framework. Soc Sci Med. 2018;203:74–80. 10.1016/j.socscimed.2017.12.026. 29305018 10.1016/j.socscimed.2017.12.026

[37] Evans CR, Leckie G, Subramanian SV, Bell A, Merlo J. A tutorial for conducting intersectional multilevel analysis of individual heterogeneity and discriminatory accuracy (MAIHDA). SSM Popul Health. 2024;26:101664. 10.1016/j.ssmph.2024.101664. 38690117 10.1016/j.ssmph.2024.101664 PMC11059336

[38] Evans CR, Erickson N. Intersectionality and depression in adolescence and early adulthood: A MAIHDA analysis of the national longitudinal study of adolescent to adult health, 1995-2008. Soc Sci Med. 2019;220:1–11. 10.1016/j.socscimed.2018.10.019. 30390469 10.1016/j.socscimed.2018.10.019

[39] Evans CR, Williams DR, Onnela J-P, Subramanian SV. A multilevel approach to modeling health inequalities at the intersection of multiple social identities. ResearchGate. 2017;203:64–73. 10.1016/j.socscimed.2017.11.011 29199054

[40] Merlo J, Wagner P, Austin PC, Subramanian SV, Leckie G. General and specific contextual effects in multilevel regression analyses and their paradoxical relationship: a conceptual tutorial. SSM Popul Health. 2018;5:33–37. 10.1016/j.ssmph.2018.05.006. 29892693 10.1016/j.ssmph.2018.05.006 PMC5993177

[41] Bell A, Evans C, Holman D, Leckie G. Extending intersectional multilevel analysis of individual heterogeneity and discriminatory accuracy (MAIHDA) to study individual longitudinal trajectories, with application to mental health in the UK. Soc Sci Med. 2024;351:116955. 10.1016/j.socscimed.2024.116955. 38762996 10.1016/j.socscimed.2024.116955

[42] UK Biobank. Learn more about UK Biobank. UK Biobank - UK Biobank. 2025. https://www.ukbiobank.ac.uk/learn-more-about-uk-biobank.

[43] Leckie G, Charlton C. runmlwin: a program to run the MLwiN multilevel modeling software from within stata. J Stat Softw. 2013;52:1–40. 10.18637/jss.v052.i11.23761062

[44] NHS. Overview Obesity. NHS choices. https://www.nhs.uk/conditions/obesity/.

[45] Evans CR, Erickson N. Intersectionality and depression in adolescence and early adulthood: a MAIHDA analysis of the national longitudinal study of adolescent to adult health, 1995-2008. Soc Sci Med. 2019;220:1 10.1016/j.socscimed.2018.10.019. 30390469 10.1016/j.socscimed.2018.10.019

[46] CDC. Risk factors for obesity. Centers for Disease Control and Prevention. 2024. https://www.cdc.gov/obesity/risk-factors/risk-factors.html.

[47] Telleria-Aramburu N, Arroyo-Izaga M. Risk factors of overweight/obesity-related lifestyles in university students: results from the EHU12/24 study. Br J Nutr. 2022;127:914–26. 10.1017/S0007114521001483. 33955337 10.1017/S0007114521001483 PMC8908003

[48] Barua P, Kibuchi E, Aktar B, Chowdhury SF, Mithu IH, Quayyum Z, et al. The effects of social determinants on children’s health outcomes in Bangladesh slums through an intersectionality lens: an application of multilevel analysis of individual heterogeneity and discriminatory accuracy (MAIHDA). PLOS Glob Public Health. 2023;3:e0001588 10.1371/journal.pgph.0001588. 36963045 10.1371/journal.pgph.0001588 PMC10022045

[49] Subramanian SV, O’Malley AJ. Modeling neighborhood effects: the futility of comparing mixed and marginal approaches. Epidemiology. 2010;21:475–8. 10.1097/EDE.0b013e3181d74a71. 20539108 10.1097/EDE.0b013e3181d74a71 PMC3085136

[50] Merlo J, Yang M, Chaix B, Lynch J, Råstam L. A brief conceptual tutorial on multilevel analysis in social epidemiology: investigating contextual phenomena in different groups of people. J Epidemiol Community Health. 2005;59:729–36. 10.1136/jech.2004.023929. 16100308 10.1136/jech.2004.023929 PMC1733145

[51] Merlo J, Chaix B, Ohlsson H, Beckman A, Johnell K, Hjerpe P, et al. A brief conceptual tutorial of multilevel analysis in social epidemiology: using measures of clustering in multilevel logistic regression to investigate contextual phenomena. J Epidemiol Community Health. 2006;60:290–7. 10.1136/jech.2004.029454. 16537344 10.1136/jech.2004.029454 PMC2566165

[52] Khalaf K, Axelsson Fisk S, Ekberg-Jansson A, Leckie G, Perez-Vicente R, Merlo J. Geographical and sociodemographic differences in discontinuation of medication for Chronic Obstructive Pulmonary Disease - A Cross-Classified Multilevel Analysis of Individual Heterogeneity and Discriminatory Accuracy (MAIHDA). Clin Epidemiol. 2020;12:783–96. 10.2147/CLEP.S247368. 32765111 10.2147/CLEP.S247368 PMC7381094

[53] Lee KX, Quek KF, Ramadas A. Dietary and lifestyle risk factors of obesity among young adults: a scoping review of observational studies. Curr Nutr Rep. 2023;12:733–43. 10.1007/s13668-023-00513-9. 38038894 10.1007/s13668-023-00513-9

[54] Wang W, Zhou H, Qi S, Yang H, Hong X. The association between physical activities combined with dietary habits and cardiovascular risk factors. Heliyon. 2024;10:e28845. 10.1016/j.heliyon.2024.e28845. 38596005 10.1016/j.heliyon.2024.e28845 PMC11002288

[55] Khan TA, Field D, Chen V, Ahmad S, Mejia SB, Kahleová H, et al. Combination of multiple low-risk lifestyle behaviors and incident type 2 diabetes: a systematic review and dose-response meta-analysis of prospective cohort studies. Diabetes Care. 2023;46:643–56. 10.2337/dc22-1024. 36812419 10.2337/dc22-1024 PMC10020027

[56] Layman DK, Evans E, Baum JI, Seyler J, Erickson DJ, Boileau RA. Dietary protein and exercise have additive effects on body composition during weight loss in adult women. J Nutr. 2005;135:1903–10. 10.1093/jn/135.8.1903. 16046715 10.1093/jn/135.8.1903

[57] Carrasquilla GD, García-Ureña M, Fall T, Sørensen TIA, Kilpeläinen TO. Mendelian randomization suggests a bidirectional, causal relationship between physical inactivity and adiposity. eLife. 2022;11:e70386. 10.7554/eLife.70386. 35254260 10.7554/eLife.70386 PMC8975550

[58] Doan T, Leach L, Doan N, Strazdins L. Causal relationship between physical activity and body weight: a maximum likelihood treatment effect model approach using australian longitudinal data. Int J Behav Med. 2024. 10.1007/s12529-024-10336-9. 10.1007/s12529-024-10336-9 39567469

