# Supplementary material for: Multilevel analysis of individual heterogeneity and discriminatory accuracy (MAIHDA) to understand how obesity risk varies according to multiple lifestyle behavior recommendations
Source: Int J Obes (Lond). 2026 Jan 7;50(4):819–29. doi: 10.1038/s41366-025-02010-1 (PMC13056532; doi:10.1038/s41366-025-02010-1)
Supplement: Supplementary file 1 — Supplementary materials [file 41366_2025_2010_MOESM1_ESM.docx]

**Supplementary figure 1.**

**Smoking**

Exclude all non-responses = 2,950

**n = 499,406**

**UK BioBank participants**

**n = 502,356**

**Physical Activity**

Exclude all non-responses = 115,195

**n = 384,211**

**Alcohol intake**

Exclude all non-responses = 112,964

**n = 271,247**

**Fruit & vegetable intake**

Exclude all non-responses = 2,455

**n = 268,792**

**Sleep**

Exclude all non-responses = 302

**n = 268,490**

**Ethnicity**

Exclude all non-responses = 645

**n = 267,845**

**TDI**

Exclude all non-responses = 303

**n = 267,542**

**Employment**

Exclude all non-responses = 1,558

**n = 265,985**

**BMI**

Exclude all non-responses = 989

**n = 264,995**

**Final sample**

**n = 264,995**

**Supplementary table 1. Biobank variables and recoding**

| Variable categories | Variables used in the study | Subcategory | Biobank Variable ID | Recoding |
| --- | --- | --- | --- | --- |
| Lifestyle variables | At or above moderate or vigorous physical activity and walking recommendations? |  | 22036 | NA |
|  | Fruit and vegetable intake | Cooked vegetable intake | 1289 | The individual fruit and vegetable variable responses were converted to portions before summing them up to obtain daily fruit and vegetable intake. To do this, we first converted heaped tablespoons (responses of both cooked and raw vegetable intake) into portions (1 portion ~ 3 heaped tablespoons) using the recommendations by previous biobank literature [1]. For fresh and dried fruit intake, we retained the responses of the variables as they are equivalent to portions. We then created 2 responses for Fruit and vegetable intake as meeting or not meeting the public health recommendation of having at least 5 potions of fruits and vegetables per day. |
|  |  | Salad/Raw vegetable intake | 1299 |  |
|  |  | Fresh fruit intake | 1309 |  |
|  |  | Dried fruit intake | 1319 |  |
|  | Alcohol intake | Average weekly red wine intake | 1568 | We converted the responses of alcohol categories into units, before summing up the responses into ‘Alcohol intake’ variable. To do this, we first converted the respective responses of red wine intake (1 unit = 2.1 glasses), champagne or white wine intake (1 unit = 2.1 glasses), beer and cider (1 unit = 2.5 pints), spirits (1 unit = 1.2 measures), fortified wine (1 unit = 3.2 glasses), and other alcoholic drinks (1 unit = 1 glass) into units [2]. This was followed by creating 2 responses as meeting or not meeting the national recommendations of limiting weekly alcohol intake to 14 units per week. |
|  |  | Average weekly champagne + white wine intake | 1578 |  |
|  |  | Average weekly beer + cider intake | 1588 |  |
|  |  | Average weekly spirits intake | 1598 |  |
|  |  | Average weekly fortified wine intake | 1608 |  |
|  |  | Average weekly intake of other alcoholic drinks | 5364 |  |
|  | Average sleeping duration per day |  | 1160 | We categorised the responses into either meeting or not meeting the public health recommendations of 7-9 hours of sleep per day. |
|  | Smoking status |  | 20116 | NA |
| Outcome variables | BMI |  | 21001 | We categorised bmi estimates into the WHO classifications of: < 18.5 kg/m^2^ (underweight), 18.5 – 24.9 kg/m^2^ (normal), 25 – 29.9 kg/m^2^ (overweight) and >30 kg/m^2^ (Obese) [3]. |
| Covariates | Age at recruitment |  | 21022 | NA |
|  | Ethnic background |  | 21000 | We recoded the ethnicity responses (white, mixed, Asian or Asian British, black or black British, Chinese, other) as ‘White British’ and ‘Non-white’ by recategorizing all original responses other than white British as non-white. |
|  | Socioeconomic position | Townsend deprivation index (TDI) | 22189 | NA |
|  |  | Employment status | 6142 | NA |

**Supplementary figure 2.**


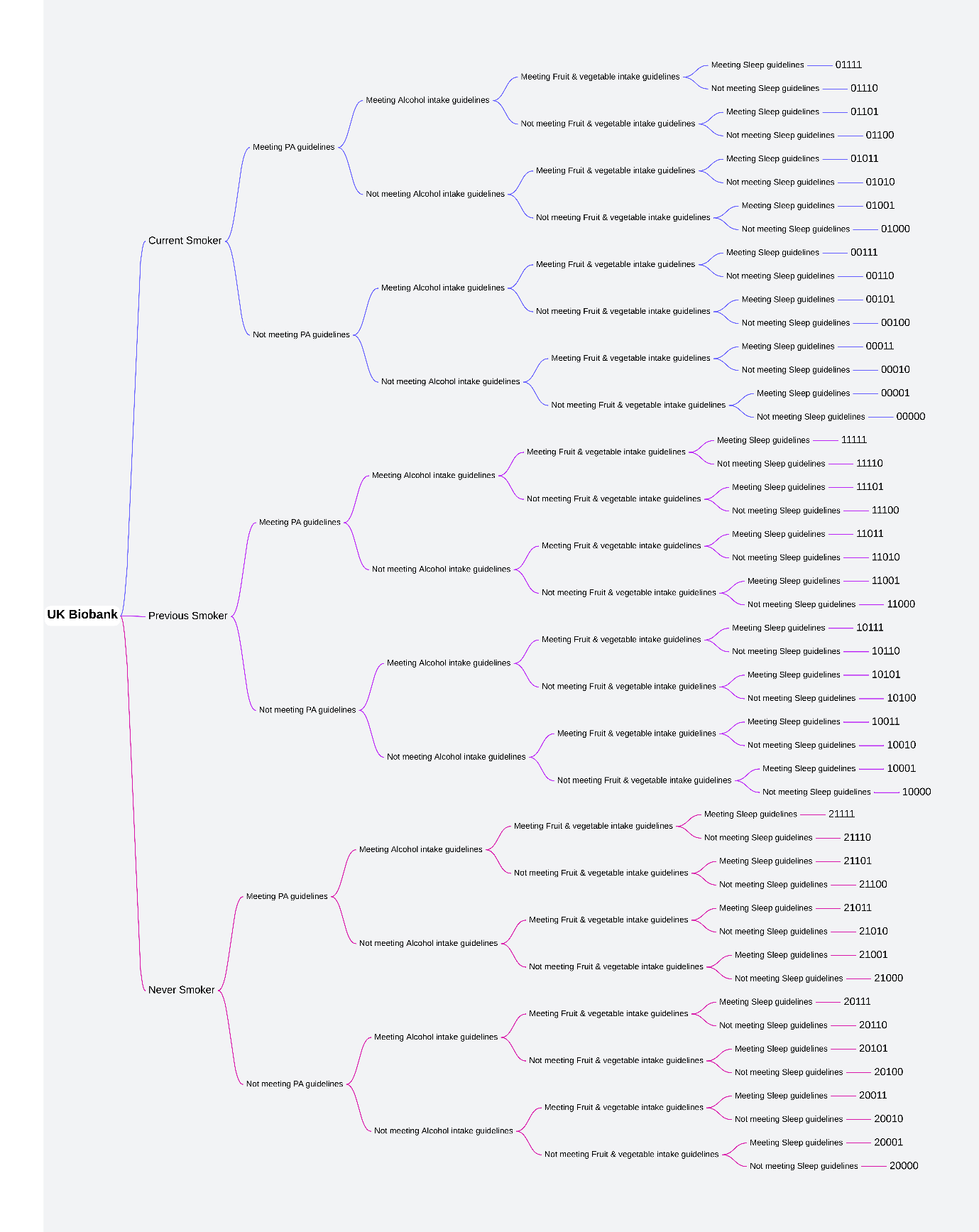


**Supplementary table 2. Strata ranked in order of their predicted mean BMIs (Males)**

| Strata ID | N | Fruit & veg | Physical activity | Sleep | Alcohol | Smoking | Observed BMI | Predicted BMI including random intercept (95% CI) | Predicted BMI excluding random intercept (95% CI) | Random intercept (95% CI) |
| --- | --- | --- | --- | --- | --- | --- | --- | --- | --- | --- |
| 11112 | 4170 | Meets | Meets | Meets | Meets | Never | 26.4 | 26.7 (26.4, 26.9) | 26.8 (26.6, 27.0) | -0.18 (-0.39, 0.02) |
| 1112 | 8952 | Not meets | Meets | Meets | Meets | Never | 26.5 | 26.7 (26.5, 27.0) | 26.7 (26.5, 26.9) | -0.002 (-0.20, 0.19) |
| 1101 | 6179 | Not meets | Meets | Meets | Not meets | Current | 26.9 | 27.0 (26.7, 27.3) | 27.1 (26.9, 27.3) | -0.09 (-0.30, 0.11) |
| 1111 | 997 | Not meets | Meets | Meets | Meets | Current | 27.0 | 27.0 (26.7, 27.4) | 26.8 (26.6, 27.0) | 0.24 (-0.02, 0.50) |
| 11102 | 8451 | Meets | Meets | Meets | Not meets | Never | 27.0 | 27.1 (26.8, 27.4) | 27.2 (27.0, 27.4) | -0.05 (-0.25, 0.14) |
|  |  |  |  |  |  |  |  |  |  |  |
| 11111 | 275 | Meets | Meets | Meets | Meets | Current | 27.2 | 27.1 (26.7, 27.5) | 26.9 (26.7, 27.1) | 0.22 (-0.11, 0.55) |
| 1001 | 2376 | Not meets | Meets | Not meets | Not meets | Current | 27.1 | 27.2 (26.9, 27.5) | 27.8 (27.5, 28.0) | -0.58 (-0.81, -0.3) |
| 11012 | 1271 | Meets | Meets | Not meets | Meets | Never | 27.1 | 27.2 (26.9, 27.6) | 27.5 (27.3, 27.7) | -0.23 (-0.48, 0.01) |
| 11101 | 1433 | Meets | Meets | Meets | Not meets | Current | 27.3 | 27.4 (27.1, 27.7) | 27.2 (27.0, 27.4) | 0.19 (-0.05, 0.44) |
| 1102 | 21076 | Not meets | Meets | Meets | Not meets | Never | 27.2 | 27.4 (27.1, 27.7) | 27.1 (26.9, 27.3) | 0.36 (0.16, 0.55) |
|  |  |  |  |  |  |  |  |  |  |  |
| 112 | 2546 | Not meets | Not meets | Meets | Meets | Never | 27.2 | 27.4 (27.1, 27.7) | 27.8 (27.6, 28.0) | -0.38 (-0.60, -0.1) |
| 1012 | 2657 | Not meets | Meets | Not meets | Meets | Never | 27.3 | 27.5 (27.2, 27.8) | 27.4 (27.2, 27.6) | 0.09 (-0.12, 0.31) |
| 1011 | 387 | Not meets | Meets | Not meets | Meets | Current | 27.6 | 27.6 (27.2, 28.0) | 27.4 (27.2, 27.7) | 0.16 (-0.15, 0.47) |
| 11011 | 110 | Meets | Meets | Not meets | Meets | Current | 27.7 | 27.6 (27.2, 28.0) | 27.5 (27.3, 27.8) | 0.06 (-0.32, 0.44) |
| 11110 | 2348 | Meets | Meets | Meets | Meets | Previous | 27.4 | 27.6 (27.3, 27.9) | 27.8 (27.6, 28.0) | -0.21 (-0.44, 0.01) |
|  |  |  |  |  |  |  |  |  |  |  |
| 111 | 299 | Not meets | Not meets | Meets | Meets | Current | 27.5 | 27.8 (27.4, 28.2) | 27.9 (27.7, 28.1) | -0.10 (-0.43, 0.22) |
| 1110 | 4471 | Not meets | Meets | Meets | Meets | Previous | 27.6 | 27.8 (27.5, 28.1) | 27.7 (27.5, 27.9) | 0.07 (-0.13, 0.28) |
| 11002 | 2505 | Meets | Meets | Not meets | Not meets | Never | 27.7 | 27.8 (27.5, 28.1) | 27.8 (27.6, 28.0) | 0.03 (-0.18, 0.26) |
| 10112 | 598 | Meets | Not meets | Meets | Meets | Never | 27.6 | 27.9 (27.5, 28.2) | 27.9 (27.7, 28.1) | -0.07 (-0.36, 0.21) |
| 11001 | 544 | Meets | Meets | Not meets | Not meets | Current | 27.9 | 27.9 (27.5, 28.3) | 27.9 (27.6, 28.1) | 0.04 (-0.25, 0.34) |
|  |  |  |  |  |  |  |  |  |  |  |
| 1100 | 20101 | Not meets | Meets | Meets | Not meets | Previous | 27.8 | 28.0 (27.7, 28.3) | 28.1 (27.9, 28.2) | -0.06 (-0.26, 0.12) |
| 101 | 1603 | Not meets | Not meets | Meets | Not meets | Current | 27.8 | 28.0 (27.7, 28.3) | 28.2 (28.0, 28.4) | -0.20 (-0.45, 0.03) |
| 1002 | 6303 | Not meets | Meets | Not meets | Not meets | Never | 27.8 | 28.0 (27.7, 28.3) | 27.7 (27.5, 27.9) | 0.30 (0.09, 0.50) |
| 11100 | 8651 | Meets | Meets | Meets | Not meets | Previous | 28.0 | 28.0 (27.8, 28.3) | 28.2 (28.0, 28.3) | -0.11 (-0.31, 0.08) |
| 10111 | 51 | Meets | Not meets | Meets | Meets | Current | 28.7 | 28.1 (27.6, 28.6) | 28.0 (27.7, 28.2) | 0.13 (-0.27, 0.54) |
|  |  |  |  |  |  |  |  |  |  |  |
| 102 | 4689 | Not meets | Not meets | Meets | Not meets | Never | 28.1 | 28.2 (27.9, 28.5) | 28.1 (27.9, 28.3) | 0.05 (-0.15, 0.26) |
| 10102 | 1000 | Meets | Not meets | Meets | Not meets | Never | 28.1 | 28.3 (27.9, 28.6) | 28.2 (28.0, 28.4) | 0.03 (-0.22, 0.29) |
| 12 | 803 | Not meets | Not meets | Not meets | Meets | Never | 28.1 | 28.3 (28.0, 28.7) | 28.5 (28.3, 28.7) | -0.15 (-0.42, 0.11) |
| 10101 | 204 | Meets | Not meets | Meets | Not meets | Current | 28.3 | 28.4 (28.0, 28.8) | 28.3 (28.1, 28.5) | 0.07 (-0.27, 0.42) |
| 1 | 792 | Not meets | Not meets | Not meets | Not meets | Current | 28.1 | 28.4 (28.1, 28.8) | 28.8 (28.6, 29.1) | -0.43 (-0.71, -0.10) |
|  |  |  |  |  |  |  |  |  |  |  |
| 11010 | 777 | Meets | Meets | Not meets | Meets | Previous | 28.3 | 28.5 (28.1, 28.8) | 28.5 (28.3, 28.7) | -0.02 (-0.29, 0.25) |
| 10012 | 229 | Meets | Not meets | Not meets | Meets | Never | 28.2 | 28.5 (28.1, 28.9) | 28.6 (28.3, 28.8) | -0.07 (-0.42, 0.26) |
| 11000 | 2712 | Meets | Meets | Not meets | Not meets | Previous | 28.3 | 28.5 (28.2, 28.8) | 28.8 (28.6, 29.0) | -0.30 (-0.52, -0.01) |
| 1000 | 6196 | Not meets | Meets | Not meets | Not meets | Previous | 28.4 | 28.6 (28.3, 28.8) | 28.7 (28.5, 28.9) | -0.13 (-0.34, 0.06) |
| 1010 | 1452 | Not meets | Meets | Not meets | Meets | Previous | 28.5 | 28.6 (28.3, 28.9) | 28.4 (28.2, 28.6) | 0.22 (-0.01, 0.46) |
|  |  |  |  |  |  |  |  |  |  |  |
| 11 | 141 | Not meets | Not meets | Not meets | Meets | Current | 28.8 | 28.6 (28.2, 29.1) | 28.5 (28.3, 28.8) | 0.12 (-0.25, 0.49) |
| 10011 | 23 | Meets | Not meets | Not meets | Meets | Current | 28.7 | 28.6 (28.1, 29.1) | 28.6 (28.4, 28.9) | 0.01 (-0.40, 0.44) |
| 110 | 1163 | Not meets | Not meets | Meets | Meets | Previous | 28.6 | 28.8 (28.4, 29.1) | 28.8 (28.6, 29.0) | -0.04 (-0.30, 0.20) |
| 10110 | 353 | Meets | Not meets | Meets | Meets | Previous | 28.6 | 28.8 (28.4, 29.2) | 28.9 (28.7, 29.1) | -0.09 (-0.41, 0.22) |
| 2 | 1674 | Not meets | Not meets | Not meets | Not meets | Never | 28.8 | 29.0 (28.7, 29.3) | 28.8 (28.6, 29.0) | 0.18 (-0.05, 0.41) |
|  |  |  |  |  |  |  |  |  |  |  |
| 10002 | 357 | Meets | Not meets | Not meets | Not meets | Never | 28.8 | 29.0 (28.6, 29.4) | 28.9 (28.7, 29.1) | 0.09 (-0.22, 0.40) |
| 10001 | 104 | Meets | Not meets | Not meets | Not meets | Current | 29.4 | 29.1 (28.7, 29.6) | 28.9 (28.7, 29.2) | 0.15 (-0.22, 0.54) |
| 100 | 4456 | Not meets | Not meets | Meets | Not meets | Previous | 29.1 | 29.2 (28.9, 29.5) | 29.1 (28.9, 29.3) | 0.03 (-0.17, 0.24) |
| 10100 | 1139 | Meets | Not meets | Meets | Not meets | Previous | 29.3 | 29.4 (29.1, 29.8) | 29.2 (29.0, 29.4) | 0.19 (-0.05, 0.45) |
| 10010 | 147 | Meets | Not meets | Not meets | Meets | Previous | 29.3 | 29.5 (29.1, 30.0) | 29.6 (29.3, 29.8) | -0.01 (-0.38, 0.35) |
|  |  |  |  |  |  |  |  |  |  |  |
| 10 | 445 | Not meets | Not meets | Not meets | Meets | Previous | 29.7 | 29.7 (29.3, 30.1) | 29.5 (29.2, 29.7) | 0.24 (-0.06, 0.54) |
| 0 | 1642 | Not meets | Not meets | Not meets | Not meets | Previous | 29.7 | 29.9 (29.6, 30.2) | 29.8 (29.6, 30.0) | 0.11 (-0.12, 0.35) |
| 10000 | 430 | Meets | Not meets | Not meets | Not meets | Previous | 30.0 | 30.0 (29.6, 30.4) | 29.9 (29.7, 30.1) | 0.12 (-0.18, 0.43) |

All estimates are from the main effects plus confounders model

**Supplementary table 3. Strata ranked in order of their predicted mean BMIs (Females)**

| Strata ID | N | Fruit & veg | Physical activity | Sleeping | Alcohol | Smoking | Observed BMI | Predicted BMI including random intercept (95% CI) | Predicted BMI excluding random intercept (95% CI) | Random intercept (95% CI) |
| --- | --- | --- | --- | --- | --- | --- | --- | --- | --- | --- |
| 11101 | 1043 | Meets | Meets | Meets | Not meets | Current | 25.5 | 25.7 (25.4, 26.0) | 25.6 (25.4, 25.8) | 0.09 (-0.14, 0.33) |
| 11102 | 8055 | Meets | Meets | Meets | Not meets | Never | 25.5 | 25.8 (25.5, 26.0) | 25.6 (25.5, 25.8) | 0.10 (-0.06, 0.27) |
| 1101 | 2847 | Not meets | Meets | Meets | Not meets | Current | 25.5 | 25.8 (25.5, 26.1) | 26.0 (25.8, 26.1) | -0.17 (-0.37, 0.02) |
| 11112 | 10660 | Meets | Meets | Meets | Meets | Never | 25.6 | 25.9 (25.6, 26.1) | 25.8 (25.6, 26.0) | 0.03 (-0.12, 0.20) |
| 11111 | 590 | Meets | Meets | Meets | Meets | Current | 26.1 | 26.0 (25.6, 26.3) | 25.8 (25.6, 25.9) | 0.20 (-0.05, 0.46) |
|  |  |  |  |  |  |  |  |  |  |  |
| 11100 | 7060 | Meets | Meets | Meets | Not meets | Previous | 25.8 | 26.0 (25.8, 26.3) | 26.1 (25.9, 26.3) | -0.07 (-0.24, 0.09) |
| 1102 | 12713 | Not meets | Meets | Meets | Not meets | Never | 25.8 | 26.1 (25.9, 26.4) | 26.0 (25.9, 26.2) | 0.10 (-0.06, 0.26) |
| 11002 | 2375 | Meets | Meets | Not meets | Not meets | Never | 26.0 | 26.2 (25.9, 26.4) | 26.2 (26.1, 26.4) | -0.06 (-0.26, 0.13) |
| 1112 | 14701 | Not meets | Meets | Meets | Meets | Never | 25.8 | 26.2 (26.0, 26.4) | 26.2 (26.1, 26.4) | -0.02 (-0.19, 0.13) |
| 1111 | 1370 | Not meets | Meets | Meets | Meets | Current | 26.1 | 26.3 (26.0, 26.6) | 26.1 (26.0, 26.3) | 0.12 (-0.10, 0.34) |
|  |  |  |  |  |  |  |  |  |  |  |
| 11011 | 223 | Meets | Meets | Not meets | Meets | Current | 26.1 | 26.3 (25.9, 26.7) | 26.3 (26.1, 26.5) | -0.02 (-0.31, 0.27) |
| 11001 | 352 | Meets | Meets | Not meets | Not meets | Current | 26.4 | 26.3 (26.0, 26.6) | 26.1 (25.9, 26.3) | 0.15 (-0.12, 0.43) |
| 11110 | 4910 | Meets | Meets | Meets | Meets | Previous | 26.1 | 26.3 (26.1, 26.6) | 26.3 (26.1, 26.4) | 0.05 (-0.12, 0.23) |
| 11000 | 2268 | Meets | Meets | Not meets | Not meets | Previous | 26.1 | 26.3 (26.1, 26.6) | 26.7 (26.5, 26.8) | -0.32 (-0.53, -0.12) |
| 11012 | 3007 | Meets | Meets | Not meets | Meets | Never | 26.2 | 26.4 (26.1, 26.6) | 26.4 (26.2, 26.5) | 0.01 (-0.18, 0.20) |
|  |  |  |  |  |  |  |  |  |  |  |
| 1001 | 1021 | Not meets | Meets | Not meets | Not meets | Current | 26.2 | 26.5 (26.2, 26.8) | 26.5 (26.4, 26.7) | -0.07 (-0.30, 0.16) |
| 1100 | 10114 | Not meets | Meets | Meets | Not meets | Previous | 26.2 | 26.5 (26.3, 26.7) | 26.5 (26.3, 26.6) | 0.003 (-0.16, 0.17) |
| 1011 | 506 | Not meets | Meets | Not meets | Meets | Current | 26.4 | 26.6 (26.3, 26.9) | 26.7 (26.5, 26.9) | -0.10 (-0.36, 0.16) |
| 1110 | 6222 | Not meets | Meets | Meets | Meets | Previous | 26.4 | 26.6 (26.4, 26.9) | 26.7 (26.5, 26.8) | -0.01 (-0.18, 0.16) |
| 1012 | 3790 | Not meets | Meets | Not meets | Meets | Never | 26.4 | 26.7 (26.4, 26.9) | 26.8 (26.6, 26.9) | -0.10 (-0.28, 0.08) |
|  |  |  |  |  |  |  |  |  |  |  |
| 11010 | 1604 | Meets | Meets | Not meets | Meets | Previous | 26.6 | 26.7 (26.5, 27.0) | 26.8 (26.7, 27.0) | -0.08 (-0.30, 0.12) |
| 1002 | 3322 | Not meets | Meets | Not meets | Not meets | Never | 26.6 | 26.8 (26.6, 27.1) | 26.6 (26.4, 26.8) | 0.19 (0.01, 0.38) |
| 1000 | 2957 | Not meets | Meets | Not meets | Not meets | Previous | 26.6 | 26.8 (26.6, 27.1) | 27.0 (26.9, 27.2) | -0.23 (-0.42, -0.04) |
| 10101 | 154 | Meets | Not meets | Meets | Not meets | Current | 26.6 | 27.1 (26.7, 27.4) | 27.1 (26.9, 27.3) | -0.02 (-0.33, 0.27) |
| 10112 | 1399 | Meets | Not meets | Meets | Meets | Never | 26.7 | 27.1 (26.8, 27.4) | 27.3 (27.2, 27.5) | -0.21 (-0.43, 0.01) |
|  |  |  |  |  |  |  |  |  |  |  |
| 10102 | 994 | Meets | Not meets | Meets | Not meets | Never | 27.0 | 27.2 (26.9, 27.5) | 27.2 (27.0, 27.3) | 0.01 (-0.21, 0.25) |
| 10111 | 89 | Meets | Not meets | Meets | Meets | Current | 27.0 | 27.3 (26.9, 27.6) | 27.3 (27.0, 27.5) | 0.01 (-0.31, 0.31) |
| 101 | 805 | Not meets | Not meets | Meets | Not meets | Current | 27.0 | 27.3 (27.0, 27.6) | 27.5 (27.3, 27.7) | -0.16 (-0.40, 0.08) |
| 112 | 3582 | Not meets | Not meets | Meets | Meets | Never | 27.0 | 27.4 (27.1, 27.6) | 27.7 (27.6, 27.9) | -0.34 (-0.52, -0.15) |
| 1010 | 1849 | Not meets | Meets | Not meets | Meets | Previous | 27.3 | 27.4 (27.2, 27.7) | 27.2 (27.1, 27.4) | 0.21 (0.000, 0.41) |
|  |  |  |  |  |  |  |  |  |  |  |
| 10001 | 76 | Meets | Not meets | Not meets | Not meets | Current | 27.2 | 27.6 (27.2, 28.0) | 27.7 (27.4, 27.9) | -0.03 (-0.35, 0.28) |
| 10100 | 904 | Meets | Not meets | Meets | Not meets | Previous | 27.5 | 27.7 (27.4, 27.9) | 27.6 (27.4, 27.8) | 0.05 (-0.18, 0.29) |
| 102 | 2963 | Not meets | Not meets | Meets | Not meets | Never | 27.3 | 27.7 (27.4, 27.9) | 27.5 (27.4, 27.7) | 0.13 (-0.05, 0.32) |
| 111 | 379 | Not meets | Not meets | Meets | Meets | Current | 27.5 | 27.7 (27.3, 28.0) | 27.7 (27.5, 27.8) | 0.02 (-0.24, 0.30) |
| 10002 | 339 | Meets | Not meets | Not meets | Not meets | Never | 27.5 | 27.7 (27.4, 28.1) | 27.7 (27.5, 27.9) | 0.01 (-0.26, 0.29) |
|  |  |  |  |  |  |  |  |  |  |  |
| 10011 | 43 | Meets | Not meets | Not meets | Meets | Current | 27.4 | 27.8 (27.4, 28.2) | 27.8 (27.6, 28.0) | -0.01 (-0.34, 0.31) |
| 10012 | 494 | Meets | Not meets | Not meets | Meets | Never | 27.5 | 27.8 (27.5, 28.1) | 27.9 (27.7, 28.1) | -0.07 (-0.34, 0.18) |
| 10110 | 713 | Meets | Not meets | Meets | Meets | Previous | 27.9 | 27.9 (27.6, 28.3) | 27.8 (27.6, 27.9) | 0.17 (-0.06, 0.42) |
| 100 | 2287 | Not meets | Not meets | Meets | Not meets | Previous | 27.6 | 28.0 (27.7, 28.2) | 28.0 (27.8, 28.2) | -0.02 (-0.23, 0.17) |
| 1 | 317 | Not meets | Not meets | Not meets | Not meets | Current | 27.8 | 28.0 (27.7, 28.4) | 28.0 (27.9, 28.2) | -0.00 (-0.29, 0.27) |
|  |  |  |  |  |  |  |  |  |  |  |
| 110 | 1514 | Not meets | Not meets | Meets | Meets | Previous | 27.8 | 28.1 (27.8, 28.4) | 28.2 (28.0, 28.3) | -0.08 (-0.30, 0.13) |
| 10000 | 324 | Meets | Not meets | Not meets | Not meets | Previous | 27.8 | 28.1 (27.8, 28.4) | 28.2 (28.0, 28.3) | -0.06 (-0.34, 0.21) |
| 12 | 1053 | Not meets | Not meets | Not meets | Meets | Never | 27.8 | 28.2 (27.9, 28.5) | 28.3 (28.1, 28.5) | -0.08 (-0.31, 0.14) |
| 11 | 176 | Not meets | Not meets | Not meets | Meets | Current | 28.1 | 28.2 (27.9, 28.6) | 28.2 (28.0, 28.4) | 0.00 (-0.29, 0.31) |
| 2 | 871 | Not meets | Not meets | Not meets | Not meets | Never | 28.2 | 28.4 (28.1, 28.7) | 28.1 (27.9, 28.3) | 0.27 (0.038, 0.51) |
|  |  |  |  |  |  |  |  |  |  |  |
| 10010 | 284 | Meets | Not meets | Not meets | Meets | Previous | 28.4 | 28.4 (28.1, 28.7) | 28.3 (28.1, 28.5) | 0.07 (-0.21, 0.35) |
| 0 | 727 | Not meets | Not meets | Not meets | Not meets | Previous | 28.5 | 28.7 (28.4, 29.0) | 28.6 (28.4, 28.7) | 0.10 (-0.14, 0.35) |
| 10 | 518 | Not meets | Not meets | Not meets | Meets | Previous | 29.1 | 29.0 (28.6, 29.3) | 28.7 (28.5, 28.9) | 0.23 (-0.03, 0.49) |

All estimates are from the main effects plus confounders model

**Supplementary table 4. Strata ranked in order of their predicted probability of obesity (Females)**

| Strata ID | N | Fruit & veg | Physical activity | Sleeping | Alcohol | Smoking | Observed probability of obesity | Predicted probability of obesity excluding random intercept (95% CI) | Predicted probability of obesity including random intercept (95% CI) | Random intercept (95% CI) |
| --- | --- | --- | --- | --- | --- | --- | --- | --- | --- | --- |
| 11101 | 1043 | Meets | Meets | Meets | Not meets | Current | 0.18 | 0.20 (0.18, 0.22) | 0.20 (0.18, 0.21) | -0.01 (-0.10, 0.10) |
| 11102 | 8055 | Meets | Meets | Meets | Not meets | Never | 0.19 | 0.21 (0.19, 0.23) | 0.21 (0.20, 0.22) | 0.01 (-0.07, 0.08) |
| 11111 | 590 | Meets | Meets | Meets | Meets | Current | 0.23 | 0.22 (0.19, 0.25) | 0.21 (0.20, 0.23) | 0.03 (-0.07, 0.14) |
| 11112 | 10660 | Meets | Meets | Meets | Meets | Never | 0.20 | 0.22 (0.21, 0.24) | 0.22 (0.21, 0.24) | 0.01 (-0.07, 0.07) |
| 1101 | 2847 | Not meets | Meets | Meets | Not meets | Current | 0.20 | 0.23 (0.21, 0.25) | 0.24 (0.23, 0.26) | -0.06 (-0.16, 0.02) |
|  |  |  |  |  |  |  |  |  |  |  |
| 11100 | 7060 | Meets | Meets | Meets | Not meets | Previous | 0.21 | 0.23 (0.21, 0.25) | 0.25 (0.23, 0.26) | -0.07 (-0.16, 0.01) |
| 11001 | 352 | Meets | Meets | Not meets | Not meets | Current | 0.29 | 0.26 (0.23, 0.29) | 0.25 (0.23, 0.27) | 0.03 (-0.07, 0.15) |
| 1102 | 12713 | Not meets | Meets | Meets | Not meets | Never | 0.23 | 0.26 (0.24, 0.28) | 0.26 (0.24, 0.27) | 0.02 (-0.05, 0.09) |
| 11002 | 2375 | Meets | Meets | Not meets | Not meets | Never | 0.24 | 0.26 (0.24, 0.29) | 0.27 (0.25, 0.28) | -0.01 (-0.09, 0.09) |
| 11011 | 223 | Meets | Meets | Not meets | Meets | Current | 0.25 | 0.27 (0.24, 0.30) | 0.27 (0.25, 0.29) | -0.01 (-0.12, 0.11) |
|  |  |  |  |  |  |  |  |  |  |  |
| 11110 | 4910 | Meets | Meets | Meets | Meets | Previous | 0.25 | 0.27 (0.25, 0.29) | 0.26 (0.25, 0.28) | 0.03 (-0.05, 0.11) |
| 1112 | 14701 | Not meets | Meets | Meets | Meets | Never | 0.23 | 0.27 (0.25, 0.29) | 0.27 (0.26, 0.28) | -0.01 (-0.08, 0.05) |
| 1111 | 1370 | Not meets | Meets | Meets | Meets | Current | 0.26 | 0.27 (0.24, 0.30) | 0.26 (0.24, 0.28) | 0.05 (-0.04, 0.15) |
| 11012 | 3007 | Meets | Meets | Not meets | Meets | Never | 0.26 | 0.28 (0.26, 0.31) | 0.28 (0.27, 0.30) | 0.01 (-0.07, 0.10) |
| 11000 | 2268 | Meets | Meets | Not meets | Not meets | Previous | 0.26 | 0.29 (0.26, 0.31) | 0.31 (0.29, 0.32) | -0.09 (-0.19, -0.00) |
|  |  |  |  |  |  |  |  |  |  |  |
| 1100 | 10114 | Not meets | Meets | Meets | Not meets | Previous | 0.27 | 0.30 (0.27, 0.32) | 0.30 (0.28, 0.31) | -0.01 (-0.07, 0.07) |
| 1001 | 1021 | Not meets | Meets | Not meets | Not meets | Current | 0.27 | 0.30 (0.27, 0.33) | 0.30 (0.28, 0.32) | -0.01 (-0.12, 0.08) |
| 1011 | 506 | Not meets | Meets | Not meets | Meets | Current | 0.30 | 0.32 (0.29, 0.35) | 0.32 (0.30, 0.34) | -0.01 (-0.12, 0.09) |
| 1110 | 6222 | Not meets | Meets | Meets | Meets | Previous | 0.29 | 0.32 (0.29, 0.34) | 0.31 (0.30, 0.33) | 0.02 (-0.05, 0.10) |
| 11010 | 1604 | Meets | Meets | Not meets | Meets | Previous | 0.30 | 0.32 (0.29, 0.35) | 0.32 (0.31, 0.34) | -0.01 (-0.11, 0.08) |
|  |  |  |  |  |  |  |  |  |  |  |
| 1002 | 3322 | Not meets | Meets | Not meets | Not meets | Never | 0.31 | 0.33 (0.31, 0.36) | 0.32 (0.30, 0.33) | 0.06 (-0.02, 0.15) |
| 1012 | 3790 | Not meets | Meets | Not meets | Meets | Never | 0.30 | 0.33 (0.31, 0.36) | 0.34 (0.32, 0.35) | -0.01 (-0.09, 0.07) |
| 1000 | 2957 | Not meets | Meets | Not meets | Not meets | Previous | 0.31 | 0.34 (0.32, 0.37) | 0.36 (0.34, 0.38) | -0.08 (-0.16, 0.01) |
| 10101 | 154 | Meets | Not meets | Meets | Not meets | Current | 0.32 | 0.35 (0.32, 0.39) | 0.35 (0.33, 0.38) | 0.01 (-0.11, 0.12) |
| 10111 | 89 | Meets | Not meets | Meets | Meets | Current | 0.37 | 0.37 (0.33, 0.41) | 0.37 (0.35, 0.40) | 0.01 (-0.11, 0.12) |
|  |  |  |  |  |  |  |  |  |  |  |
| 10102 | 994 | Meets | Not meets | Meets | Not meets | Never | 0.35 | 0.38 (0.35, 0.41) | 0.37 (0.35, 0.39) | 0.03 (-0.06, 0.14) |
| 10112 | 1399 | Meets | Not meets | Meets | Meets | Never | 0.33 | 0.38 (0.35, 0.41) | 0.39 (0.37, 0.41) | -0.03 (-0.13, 0.06) |
| 101 | 805 | Not meets | Not meets | Meets | Not meets | Current | 0.35 | 0.41 (0.37, 0.44) | 0.41 (0.39, 0.44) | -0.03 (-0.13, 0.07) |
| 1010 | 1849 | Not meets | Meets | Not meets | Meets | Previous | 0.41 | 0.41 (0.38, 0.44) | 0.38 (0.36, 0.40) | 0.10 (0.014, 0.20) |
| 112 | 3582 | Not meets | Not meets | Meets | Meets | Never | 0.36 | 0.42 (0.40, 0.45) | 0.45 (0.43, 0.47) | -0.10 (-0.19, -0.02) |
|  |  |  |  |  |  |  |  |  |  |  |
| 10100 | 904 | Meets | Not meets | Meets | Not meets | Previous | 0.41 | 0.43 (0.39, 0.46) | 0.42 (0.40, 0.44) | 0.03 (-0.07, 0.13) |
| 10001 | 76 | Meets | Not meets | Not meets | Not meets | Current | 0.43 | 0.43 (0.39, 0.47) | 0.43 (0.40, 0.45) | 0.01 (-0.11, 0.12) |
| 111 | 379 | Not meets | Not meets | Meets | Meets | Current | 0.39 | 0.43 (0.40, 0.47) | 0.43 (0.41, 0.46) | -0.01 (-0.11, 0.11) |
| 102 | 2963 | Not meets | Not meets | Meets | Not meets | Never | 0.39 | 0.44 (0.41, 0.47) | 0.43 (0.41, 0.45) | 0.04 (-0.04, 0.13) |
| 10002 | 339 | Meets | Not meets | Not meets | Not meets | Never | 0.42 | 0.44 (0.41, 0.48) | 0.44 (0.42, 0.47) | 0.01 (-0.10, 0.12) |
|  |  |  |  |  |  |  |  |  |  |  |
| 10011 | 43 | Meets | Not meets | Not meets | Meets | Current | 0.40 | 0.44 (0.40, 0.49) | 0.45 (0.42, 0.47) | -0.01 (-0.12, 0.12) |
| 10110 | 713 | Meets | Not meets | Meets | Meets | Previous | 0.45 | 0.45 (0.42, 0.48) | 0.44 (0.42, 0.46) | 0.04 (-0.05, 0.15) |
| 10012 | 494 | Meets | Not meets | Not meets | Meets | Never | 0.38 | 0.45 (0.41, 0.49) | 0.46 (0.44, 0.49) | -0.04 (-0.15, 0.06) |
| 100 | 2287 | Not meets | Not meets | Meets | Not meets | Previous | 0.45 | 0.49 (0.46, 0.52) | 0.48 (0.46, 0.50) | 0.03 (-0.06, 0.12) |
| 110 | 1514 | Not meets | Not meets | Meets | Meets | Previous | 0.45 | 0.49 (0.46, 0.52) | 0.50 (0.48, 0.52) | -0.03 (-0.12, 0.06) |
|  |  |  |  |  |  |  |  |  |  |  |
| 1 | 317 | Not meets | Not meets | Not meets | Not meets | Current | 0.49 | 0.49 (0.46, 0.53) | 0.49 (0.46, 0.51) | 0.02 (-0.09, 0.13) |
| 10000 | 324 | Meets | Not meets | Not meets | Not meets | Previous | 0.50 | 0.50 (0.46, 0.53) | 0.49 (0.47, 0.51) | 0.01 (-0.09, 0.13) |
| 11 | 176 | Not meets | Not meets | Not meets | Meets | Current | 0.45 | 0.50 (0.47, 0.54) | 0.51 (0.48, 0.53) | -0.01 (-0.13, 0.10) |
| 10010 | 284 | Meets | Not meets | Not meets | Meets | Previous | 0.48 | 0.51 (0.47, 0.55) | 0.51 (0.49, 0.53) | -0.01 (-0.12, 0.11) |
| 2 | 871 | Not meets | Not meets | Not meets | Not meets | Never | 0.48 | 0.51 (0.48, 0.55) | 0.50 (0.48, 0.52) | 0.038 (-0.06, 0.14) |
|  |  |  |  |  |  |  |  |  |  |  |
| 12 | 1053 | Not meets | Not meets | Not meets | Meets | Never | 0.46 | 0.52 (0.48, 0.55) | 0.52 (0.50, 0.55) | -0.03 (-0.13, 0.06) |
| 0 | 727 | Not meets | Not meets | Not meets | Not meets | Previous | 0.53 | 0.56 (0.52, 0.59) | 0.55 (0.53, 0.57) | 0.01 (-0.10, 0.11) |
| 10 | 518 | Not meets | Not meets | Not meets | Meets | Previous | 0.55 | 0.58 (0.54, 0.61) | 0.57 (0.55, 0.59) | 0.01 (-0.10, 0.11) |

All estimates are from the main effects plus confounders model

**Supplementary table 5. Strata ranked in order of their predicted probability of obesity (Males)**

| Strata ID | N | Fruit & veg | Physical activity | Sleeping | Alcohol | Smoking | Observed probability of obesity | Predicted probability of obesity excluding random intercept (95% CI) | Predicted probability of obesity including random intercept (95% CI) | Random intercept (95% CI) |
| --- | --- | --- | --- | --- | --- | --- | --- | --- | --- | --- |
| 11112 | 4170 | Meets | Meets | Meets | Meets | Never | 0.28 | 0.33 (0.29, 0.37) | 0.37 (0.34, 0.39) | -0.15 (-0.29, -0.01) |
| 1112 | 8952 | Not meets | Meets | Meets | Meets | Never | 0.29 | 0.34 (0.31, 0.38) | 0.36 (0.34, 0.39) | -0.08 (-0.21, 0.03) |
| 11111 | 275 | Meets | Meets | Meets | Meets | Current | 0.40 | 0.40 (0.34, 0.47) | 0.38 (0.34, 0.41) | 0.11 (-0.09, 0.32) |
| 1111 | 997 | Not meets | Meets | Meets | Meets | Current | 0.37 | 0.41 (0.36, 0.46) | 0.37 (0.34, 0.40) | 0.14 (-0.03, 0.31) |
| 1101 | 6179 | Not meets | Meets | Meets | Not meets | Current | 0.35 | 0.41 (0.37, 0.45) | 0.43 (0.41, 0.47) | -0.10 (-0.24, 0.02) |
|  |  |  |  |  |  |  |  |  |  |  |
| 11102 | 8451 | Meets | Meets | Meets | Not meets | Never | 0.35 | 0.41 (0.37, 0.45) | 0.43 (0.40, 0.46) | -0.07 (-0.20, 0.04) |
| 11012 | 1271 | Meets | Meets | Not meets | Meets | Never | 0.35 | 0.42 (0.37, 0.47) | 0.45 (0.42, 0.49) | -0.13 (-0.29, 0.02) |
| 1001 | 2376 | Not meets | Meets | Not meets | Not meets | Current | 0.38 | 0.44 (0.40, 0.49) | 0.52 (0.49, 0.56) | -0.32 (-0.46, -0.1) |
| 1102 | 21076 | Not meets | Meets | Meets | Not meets | Never | 0.40 | 0.46 (0.42, 0.51) | 0.43 (0.40, 0.46) | 0.15 (0.030, 0.27) |
| 112 | 2546 | Not meets | Not meets | Meets | Meets | Never | 0.39 | 0.46 (0.42, 0.51) | 0.52 (0.49, 0.55) | -0.21 (-0.35, -0.07) |
|  |  |  |  |  |  |  |  |  |  |  |
| 1012 | 2657 | Not meets | Meets | Not meets | Meets | Never | 0.41 | 0.46 (0.42, 0.51) | 0.45 (0.42, 0.48) | 0.05 (-0.08, 0.20) |
| 11101 | 1433 | Meets | Meets | Meets | Not meets | Current | 0.42 | 0.47 (0.41, 0.52) | 0.44 (0.40, 0.47) | 0.11 (-0.04, 0.27) |
| 11011 | 110 | Meets | Meets | Not meets | Meets | Current | 0.45 | 0.47 (0.40, 0.54) | 0.46 (0.43, 0.50) | 0.03 (-0.20, 0.26) |
| 1011 | 387 | Not meets | Meets | Not meets | Meets | Current | 0.46 | 0.48 (0.42, 0.54) | 0.46 (0.43, 0.49) | 0.10 (-0.09, 0.29) |
| 11110 | 2348 | Meets | Meets | Meets | Meets | Previous | 0.46 | 0.51 (0.46, 0.56) | 0.53 (0.50, 0.56) | -0.06 (-0.21, 0.07) |
|  |  |  |  |  |  |  |  |  |  |  |
| 11002 | 2505 | Meets | Meets | Not meets | Not meets | Never | 0.46 | 0.52 (0.47, 0.57) | 0.52 (0.49, 0.55) | 0.01 (-0.13, 0.14) |
| 111 | 299 | Not meets | Not meets | Meets | Meets | Current | 0.47 | 0.52 (0.46, 0.59) | 0.53 (0.49, 0.56) | -0.01 (-0.21, 0.20) |
| 10112 | 598 | Meets | Not meets | Meets | Meets | Never | 0.47 | 0.53 (0.47, 0.58) | 0.52 (0.49, 0.55) | 0.02 (-0.15, 0.21) |
| 11001 | 544 | Meets | Meets | Not meets | Not meets | Current | 0.50 | 0.54 (0.48, 0.59) | 0.53 (0.49, 0.56) | 0.04 (-0.14, 0.22) |
| 10111 | 51 | Meets | Not meets | Meets | Meets | Current | 0.57 | 0.54 (0.47, 0.61) | 0.53 (0.49, 0.57) | 0.03 (-0.20, 0.28) |
|  |  |  |  |  |  |  |  |  |  |  |
| 1110 | 4471 | Not meets | Meets | Meets | Meets | Previous | 0.49 | 0.54 (0.50, 0.59) | 0.53 (0.50, 0.56) | 0.07 (-0.06, 0.20) |
| 1002 | 6303 | Not meets | Meets | Not meets | Not meets | Never | 0.51 | 0.56 (0.52, 0.60) | 0.51 (0.48, 0.54) | 0.19 (0.06, 0.32) |
| 1100 | 20101 | Not meets | Meets | Meets | Not meets | Previous | 0.52 | 0.58 (0.53, 0.62) | 0.59 (0.56, 0.62) | -0.05 (-0.17, 0.06) |
| 101 | 1603 | Not meets | Not meets | Meets | Not meets | Current | 0.52 | 0.58 (0.53, 0.63) | 0.59 (0.56, 0.62) | -0.04 (-0.20, 0.10) |
| 11100 | 8651 | Meets | Meets | Meets | Not meets | Previous | 0.53 | 0.59 (0.54, 0.63) | 0.59 (0.56, 0.62) | -0.03 (-0.15, 0.09) |
|  |  |  |  |  |  |  |  |  |  |  |
| 12 | 803 | Not meets | Not meets | Not meets | Meets | Never | 0.52 | 0.59 (0.54, 0.64) | 0.60 (0.57, 0.63) | -0.05 (-0.22, 0.12) |
| 10101 | 204 | Meets | Not meets | Meets | Not meets | Current | 0.54 | 0.59 (0.53, 0.65) | 0.59 (0.56, 0.63) | 0.01 (-0.21, 0.22) |
| 102 | 4689 | Not meets | Not meets | Meets | Not meets | Never | 0.53 | 0.59 (0.55, 0.64) | 0.58 (0.55, 0.61) | 0.05 (-0.07, 0.19) |
| 10102 | 1000 | Meets | Not meets | Meets | Not meets | Never | 0.56 | 0.61 (0.55, 0.65) | 0.58 (0.55, 0.61) | 0.09 (-0.07, 0.26) |
| 10012 | 229 | Meets | Not meets | Not meets | Meets | Never | 0.55 | 0.61 (0.55, 0.67) | 0.61 (0.57, 0.64) | 0.01 (-0.20, 0.21) |
|  |  |  |  |  |  |  |  |  |  |  |
| 11010 | 777 | Meets | Meets | Not meets | Meets | Previous | 0.56 | 0.61 (0.56, 0.66) | 0.62 (0.59, 0.65) | -0.02 (-0.19, 0.15) |
| 10011 | 23 | Meets | Not meets | Not meets | Meets | Current | 0.53 | 0.61 (0.54, 0.68) | 0.62 (0.58, 0.65) | -0.01 (-0.25, 0.24) |
| 1 | 792 | Not meets | Not meets | Not meets | Not meets | Current | 0.54 | 0.62 (0.57, 0.67) | 0.67 (0.64, 0.70) | -0.23 (-0.40, -0.05) |
| 11 | 141 | Not meets | Not meets | Not meets | Meets | Current | 0.64 | 0.63 (0.57, 0.69) | 0.61 (0.58, 0.65) | 0.08 (-0.14, 0.30) |
| 110 | 1163 | Not meets | Not meets | Meets | Meets | Previous | 0.59 | 0.65 (0.60, 0.69) | 0.67 (0.65, 0.70) | -0.11 (-0.27, 0.05) |
|  |  |  |  |  |  |  |  |  |  |  |
| 1010 | 1452 | Not meets | Meets | Not meets | Meets | Previous | 0.62 | 0.65 (0.61, 0.70) | 0.61 (0.58, 0.64) | 0.17 (0.01, 0.33) |
| 11000 | 2712 | Meets | Meets | Not meets | Not meets | Previous | 0.60 | 0.65 (0.61, 0.69) | 0.67 (0.65, 0.70) | -0.09 (-0.23, 0.04) |
| 1000 | 6196 | Not meets | Meets | Not meets | Not meets | Previous | 0.61 | 0.66 (0.63, 0.70) | 0.67 (0.65, 0.70) | -0.03 (-0.16, 0.09) |
| 10110 | 353 | Meets | Not meets | Meets | Meets | Previous | 0.61 | 0.67 (0.61, 0.72) | 0.68 (0.64, 0.71) | -0.03 (-0.23, 0.17) |
| 10002 | 357 | Meets | Not meets | Not meets | Not meets | Never | 0.64 | 0.68 (0.62, 0.73) | 0.67 (0.64, 0.69) | 0.04 (-0.15, 0.25) |
|  |  |  |  |  |  |  |  |  |  |  |
| 2 | 1674 | Not meets | Not meets | Not meets | Not meets | Never | 0.63 | 0.68 (0.64, 0.72) | 0.66 (0.64, 0.69) | 0.08 (-0.06, 0.23) |
| 10001 | 104 | Meets | Not meets | Not meets | Not meets | Current | 0.69 | 0.68 (0.62, 0.74) | 0.67 (0.64, 0.71) | 0.04 (-0.19, 0.28) |
| 100 | 4456 | Not meets | Not meets | Meets | Not meets | Previous | 0.69 | 0.74 (0.70, 0.77) | 0.73 (0.70, 0.75) | 0.04 (-0.08, 0.18) |
| 10010 | 147 | Meets | Not meets | Not meets | Meets | Previous | 0.67 | 0.74 (0.68, 0.79) | 0.75 (0.72, 0.78) | -0.04 (-0.27, 0.18) |
| 10100 | 1139 | Meets | Not meets | Meets | Not meets | Previous | 0.73 | 0.76 (0.72, 0.79) | 0.73 (0.71, 0.75) | 0.13 (-0.03, 0.30) |
|  |  |  |  |  |  |  |  |  |  |  |
| 10 | 445 | Not meets | Not meets | Not meets | Meets | Previous | 0.74 | 0.76 (0.72, 0.80) | 0.75 (0.72, 0.77) | 0.09 (-0.10, 0.29) |
| 10000 | 430 | Meets | Not meets | Not meets | Not meets | Previous | 0.74 | 0.79 (0.75, 0.82) | 0.79 (0.77, 0.81) | -0.03 (-0.23, 0.16) |
| 0 | 1642 | Not meets | Not meets | Not meets | Not meets | Previous | 0.76 | 0.79 (0.76, 0.82) | 0.79 (0.77, 0.81) | 0.01 (-0.14, 0.17) |

All estimates are from the main effects plus confounders model

**Supplementary table 6. MAIHDA models for overweight vs normal weight**

|  | Males | | | Females | | |
| --- | --- | --- | --- | --- | --- | --- |
|  | Null Model | Main effects model | Main effects model  plus confounders^a^ | Null Model | Main effects Model | Main effects Model  plus confounders^a^ |
|  | OR (95% CI) | OR (95% CI) | OR (95% CI) | OR (95% CI) | OR (95% CI) | OR (95% CI) |
| Intercept | 2.05 (1.89, 2.22) | 3.44 (3.12, 3.80) | 4.25 (3.77, 4.78) | 0.91 (0.86, 0.96) | 1.27 (1.20, 1.34) | 1.40 (1.29, 1.52) |
| Smoking |  |  |  |  |  |  |
| Previous |  | -- | -- |  | -- | -- |
| Current |  | 0.64 (0.58, 0.71) | 0.68 (0.62, 0.75) |  | 0.84 (0.80, 0.89) | 0.89 (0.85, 0.94) |
| Never |  | 0.70 (0.64, 0.76) | 0.70 (0.65, 0.76) |  | 0.83 (0.80, 0.86) | 0.86 (0.84, 0.89) |
| Fruit & vegetable |  |  |  |  |  |  |
| Not meets |  | -- | -- |  | -- | -- |
| Meets |  | 0.96 (0.89, 1.04) | 0.96 (0.89, 1.03) |  | 0.93 (0.90, 0.96) | 0.88 (0.85, 0.90) |
| Physical activity |  |  |  |  |  |  |
| Not meets |  | -- | -- |  | -- | -- |
| Meets |  | 0.81 (0.75, 0.88) | 0.82 (0.76, 0.88) |  | 0.77 (0.74, 0.80) | 0.75 (0.73, 0.78) |
| Alcohol |  |  |  |  |  |  |
| Not meets |  | -- | -- |  | -- | -- |
| Meets |  | 0.79 (0.73, 0.85) | 0.78 (0.73, 0.84) |  | 1.02 (0.98, 1.05) | 1.01 (0.98, 1.04) |
| Sleep |  |  |  |  |  |  |
| Not meets |  | -- |  |  | -- |  |
| Meets |  | 0.91 (0.85, 0.98) | 0.90 (0.84, 0.97) |  | 0.89 (0.86, 0.93) | 0.92 (0.89, 0.95) |
| Level 2 variance | 0.06 (0.03, 0.09) | 0.009 (0.003, 0.01) | 0.009 (0.002, 0.01) | 0.02 (0.01, 0.04) | 0.0008 (-0.0002, 0.001) | 0.00008 (-0.0004, 0.0006) |
| Level 1 variance | -- | -- | -- | -- | -- | -- |
| VPC | 1.98% | 0.30% | 0.27% | 0.87% | 0.02% | 0.002% |
| PCV^b^ | -- | 85.04% | 86.45% | -- | 97.16% | 99.70% |

^a^Confounders were Age, ethnicity, tdi and employment status

^b^All estimated PCVs are relative to the null model.

**Supplementary table 7. Strata ranked in order of their predicted probability of overweight (Females)**

| Strata ID | N | Fruit & veg | Physical activity | Sleeping | Alcohol | Smoking | Observed probability of overweight | Predicted probability of overweight excluding random intercept (95% CI) | Predicted probability of overweight including random intercept (95% CI) | Random intercept  (95% CI) |
| --- | --- | --- | --- | --- | --- | --- | --- | --- | --- | --- |
| 11102 | 8055 | Meets | Meets | Meets | Not meets | Never | 0.41 | 0.43 (0.42, 0.44) | 0.43 (0.42, 0.44) | 0.001 (-0.01, 0.01) |
| 11112 | 10660 | Meets | Meets | Meets | Meets | Never | 0.41 | 0.43 (0.42, 0.44) | 0.43 (0.42, 0.44) | 0.001 (-0.01, 0.01) |
| 11101 | 1043 | Meets | Meets | Meets | Not meets | Current | 0.42 | 0.43 (0.42, 0.45) | 0.43 (0.42, 0.45) | 0.001 (-0.01, 0.01) |
| 11111 | 590 | Meets | Meets | Meets | Meets | Current | 0.42 | 0.44 (0.42, 0.45) | 0.44 (0.42, 0.45) | 0.0006 (-0.01, 0.01) |
| 11002 | 2375 | Meets | Meets | Not meets | Not meets | Never | 0.43 | 0.44 (0.43, 0.46) | 0.44 (0.44, 0.45) | 0.0005 (-0.01, 0.01) |
|  |  |  |  |  |  |  |  |  |  |  |
| 11012 | 3007 | Meets | Meets | Not meets | Meets | Never | 0.44 | 0.45 (0.44, 0.46) | 0.45 (0.44, 0.46) | 0.0009 (-0.01, 0.01) |
| 11001 | 352 | Meets | Meets | Not meets | Not meets | Current | 0.42 | 0.45 (0.44, 0.47) | 0.45 (0.44, 0.47) | -0.00009 (-0.01, 0.01) |
| 11011 | 223 | Meets | Meets | Not meets | Meets | Current | 0.42 | 0.46 (0.44, 0.47) | 0.46 (0.44, 0.47) | -0.0001 (-0.01, 0.01) |
| 1102 | 12713 | Not meets | Meets | Meets | Not meets | Never | 0.42 | 0.46 (0.45, 0.47) | 0.46 (0.45, 0.46) | -0.0002 (-0.01, 0.01) |
| 1112 | 14701 | Not meets | Meets | Meets | Meets | Never | 0.42 | 0.46 (0.45, 0.47) | 0.46 (0.45, 0.47) | -0.001 (-0.01, 0.01) |
|  |  |  |  |  |  |  |  |  |  |  |
| 11100 | 7060 | Meets | Meets | Meets | Not meets | Previous | 0.44 | 0.46 (0.45, 0.47) | 0.46 (0.45, 0.47) | -0.004 (-0.02, 0.01) |
| 1101 | 2847 | Not meets | Meets | Meets | Not meets | Current | 0.40 | 0.46 (0.45, 0.48) | 0.46 (0.45, 0.48) | -0.004 (-0.02, 0.01) |
| 11110 | 4910 | Meets | Meets | Meets | Meets | Previous | 0.46 | 0.46 (0.45, 0.48) | 0.46 (0.45, 0.48) | 0.001 (-0.01, 0.01) |
| 1111 | 1370 | Not meets | Meets | Meets | Meets | Current | 0.45 | 0.47 (0.45, 0.48) | 0.47 (0.45, 0.48) | 0.001 (-0.01, 0.01) |
| 1002 | 3322 | Not meets | Meets | Not meets | Not meets | Never | 0.46 | 0.48 (0.46, 0.49) | 0.48 (0.47, 0.49) | 0.001 (-0.01, 0.01) |
|  |  |  |  |  |  |  |  |  |  |  |
| 11000 | 2268 | Meets | Meets | Not meets | Not meets | Previous | 0.44 | 0.48 (0.47, 0.49) | 0.48 (0.47, 0.49) | -0.005 (-0.02, 0.01) |
| 1012 | 3790 | Not meets | Meets | Not meets | Meets | Never | 0.46 | 0.48 (0.47, 0.49) | 0.48 (0.47, 0.49) | 0.0007 (-0.01, 0.01) |
| 11010 | 1604 | Meets | Meets | Not meets | Meets | Previous | 0.47 | 0.48 (0.47, 0.49) | 0.48 (0.47, 0.49) | -0.0008 (-0.01, 0.01) |
| 1001 | 1021 | Not meets | Meets | Not meets | Not meets | Current | 0.45 | 0.48 (0.47, 0.50) | 0.48 (0.47, 0.50) | 0.0007 (-0.01, 0.01) |
| 1011 | 506 | Not meets | Meets | Not meets | Meets | Current | 0.43 | 0.49 (0.47, 0.50) | 0.49 (0.47, 0.50) | -0.001 (-0.01, 0.01) |
|  |  |  |  |  |  |  |  |  |  |  |
| 1100 | 10114 | Not meets | Meets | Meets | Not meets | Previous | 0.47 | 0.49 (0.48, 0.50) | 0.49 (0.48, 0.50) | 0.003 (-0.01, 0.02) |
| 10102 | 994 | Meets | Not meets | Meets | Not meets | Never | 0.49 | 0.49 (0.48, 0.51) | 0.49 (0.48, 0.51) | 0.001 (-0.01, 0.01) |
| 1110 | 6222 | Not meets | Meets | Meets | Meets | Previous | 0.47 | 0.50 (0.48, 0.51) | 0.50 (0.49, 0.51) | -0.0002 (-0.01, 0.01) |
| 10112 | 1399 | Meets | Not meets | Meets | Meets | Never | 0.45 | 0.50 (0.49, 0.51) | 0.50 (0.49, 0.51) | -0.001 (-0.01, 0.01) |
| 10101 | 154 | Meets | Not meets | Meets | Not meets | Current | 0.42 | 0.50 (0.49, 0.52) | 0.50 (0.49, 0.52) | -0.0003 (-0.01, 0.01) |
|  |  |  |  |  |  |  |  |  |  |  |
| 10111 | 89 | Meets | Not meets | Meets | Meets | Current | 0.50 | 0.51 (0.49, 0.53) | 0.51 (0.49, 0.53) | 0.0002 (-0.01, 0.01) |
| 1000 | 2957 | Not meets | Meets | Not meets | Not meets | Previous | 0.49 | 0.51 (0.50, 0.52) | 0.51 (0.50, 0.52) | -0.0006 (-0.01, 0.01) |
| 10002 | 339 | Meets | Not meets | Not meets | Not meets | Never | 0.55 | 0.51 (0.50, 0.53) | 0.51 (0.50, 0.53) | 0.001 (-0.01, 0.01) |
| 1010 | 1849 | Not meets | Meets | Not meets | Meets | Previous | 0.53 | 0.52 (0.50, 0.53) | 0.51 (0.50, 0.53) | 0.003 (-0.01, 0.02) |
| 10012 | 494 | Meets | Not meets | Not meets | Meets | Never | 0.47 | 0.52 (0.50, 0.53) | 0.52 (0.50, 0.53) | -0.0006 (-0.01, 0.01) |
|  |  |  |  |  |  |  |  |  |  |  |
| 10001 | 76 | Meets | Not meets | Not meets | Not meets | Current | 0.37 | 0.52 (0.50, 0.54) | 0.52 (0.50, 0.54) | -0.0005 (-0.01, 0.01) |
| 10011 | 43 | Meets | Not meets | Not meets | Meets | Current | 0.50 | 0.53 (0.51, 0.54) | 0.53 (0.51, 0.54) | -0.00004 (-0.01, 0.01) |
| 102 | 2963 | Not meets | Not meets | Meets | Not meets | Never | 0.48 | 0.53 (0.51, 0.54) | 0.53 (0.52, 0.54) | -0.00004 (-0.01, 0.01) |
| 112 | 3582 | Not meets | Not meets | Meets | Meets | Never | 0.46 | 0.53 (0.52, 0.54) | 0.53 (0.52, 0.54) | -0.005 (-0.02, 0.01) |
| 10100 | 904 | Meets | Not meets | Meets | Not meets | Previous | 0.54 | 0.53 (0.52, 0.54) | 0.53 (0.52, 0.54) | 0.001 (-0.01, 0.01) |
|  |  |  |  |  |  |  |  |  |  |  |
| 10110 | 713 | Meets | Not meets | Meets | Meets | Previous | 0.54 | 0.53 (0.52, 0.55) | 0.53 (0.52, 0.55) | 0.001 (-0.01, 0.01) |
| 101 | 805 | Not meets | Not meets | Meets | Not meets | Current | 0.50 | 0.53 (0.52, 0.55) | 0.53 (0.52, 0.55) | 0.0005 (-0.01, 0.01) |
| 111 | 379 | Not meets | Not meets | Meets | Meets | Current | 0.54 | 0.54 (0.52, 0.55) | 0.54 (0.52, 0.55) | 0.001 (-0.01, 0.01) |
| 2 | 871 | Not meets | Not meets | Not meets | Not meets | Never | 0.51 | 0.54 (0.53, 0.56) | 0.54 (0.53, 0.56) | 0.0001 (-0.01, 0.01) |
| 12 | 1053 | Not meets | Not meets | Not meets | Meets | Never | 0.51 | 0.55 (0.53, 0.56) | 0.55 (0.54, 0.56) | -0.00009 (-0.01, 0.01) |
|  |  |  |  |  |  |  |  |  |  |  |
| 10000 | 324 | Meets | Not meets | Not meets | Not meets | Previous | 0.58 | 0.55 (0.54, 0.56) | 0.55 (0.54, 0.56) | 0.0008 (-0.01, 0.01) |
| 10010 | 284 | Meets | Not meets | Not meets | Meets | Previous | 0.56 | 0.55 (0.54, 0.57) | 0.55 (0.54, 0.56) | 0.0001 (-0.01, 0.01) |
| 1 | 317 | Not meets | Not meets | Not meets | Not meets | Current | 0.50 | 0.55 (0.54, 0.57) | 0.55 (0.54, 0.57) | -0.00004 (-0.01, 0.01) |
| 11 | 176 | Not meets | Not meets | Not meets | Meets | Current | 0.53 | 0.56 (0.54, 0.57) | 0.56 (0.54, 0.57) | -0.00001 (-0.01, 0.01) |
| 100 | 2287 | Not meets | Not meets | Meets | Not meets | Previous | 0.54 | 0.56 (0.55, 0.57) | 0.56 (0.55, 0.57) | 0.001 (-0.01, 0.01) |
|  |  |  |  |  |  |  |  |  |  |  |
| 110 | 1514 | Not meets | Not meets | Meets | Meets | Previous | 0.53 | 0.56 (0.55, 0.58) | 0.56 (0.55, 0.58) | -0.0008 (-0.01, 0.01) |
| 0 | 727 | Not meets | Not meets | Not meets | Not meets | Previous | 0.55 | 0.58 (0.57, 0.59) | 0.58 (0.57, 0.59) | -0.0002 (-0.01, 0.01) |
| 10 | 518 | Not meets | Not meets | Not meets | Meets | Previous | 0.54 | 0.58 (0.57, 0.60) | 0.58 (0.57, 0.60) | -0.0006 (-0.01, 0.01) |

All estimates are from the main effects plus confounders model

**Supplementary table 8. Strata ranked in order of their predicted probability of overweight (Males)**

| Strata ID | N | Fruit & veg | Physical activity | Sleeping | Alcohol | Smoking | Observed probability of overweight | Predicted probability of overweight excluding random intercept (95% CI) | Predicted probability of overweight including random intercept (95% CI) | Random intercept  (95% CI) |
| --- | --- | --- | --- | --- | --- | --- | --- | --- | --- | --- |
| 11112 | 4170 | Meets | Meets | Meets | Meets | Never | 0.54 | 0.60 (0.56, 0.63) | 0.62 (0.60, 0.64) | -0.12 (-0.22, -0.02) |
| 1112 | 8952 | Not meets | Meets | Meets | Meets | Never | 0.57 | 0.62 (0.59, 0.65) | 0.63 (0.61, 0.65) | -0.07 (-0.16, 0.02) |
| 11012 | 1271 | Meets | Meets | Not meets | Meets | Never | 0.55 | 0.62 (0.58, 0.65) | 0.65 (0.63, 0.67) | -0.12 (-0.24, -0.002) |
| 11111 | 275 | Meets | Meets | Meets | Meets | Current | 0.57 | 0.62 (0.58, 0.67) | 0.62 (0.59, 0.64) | 0.02 (-0.13, 0.18) |
| 112 | 2546 | Not meets | Not meets | Meets | Meets | Never | 0.58 | 0.64 (0.61, 0.67) | 0.68 (0.66, 0.70) | -0.17 (-0.28, -0.06) |
|  |  |  |  |  |  |  |  |  |  |  |
| 11011 | 110 | Meets | Meets | Not meets | Meets | Current | 0.63 | 0.65 (0.60, 0.69) | 0.64 (0.61, 0.67) | 0.03 (-0.14, 0.20) |
| 1111 | 997 | Not meets | Meets | Meets | Meets | Current | 0.60 | 0.65 (0.61, 0.68) | 0.63 (0.60, 0.65) | 0.08 (-0.03, 0.21) |
| 1001 | 2376 | Not meets | Meets | Not meets | Not meets | Current | 0.58 | 0.66 (0.62, 0.69) | 0.70 (0.68, 0.72) | -0.19 (-0.30, -0.08) |
| 1011 | 387 | Not meets | Meets | Not meets | Meets | Current | 0.62 | 0.66 (0.62, 0.70) | 0.65 (0.63, 0.67) | 0.06 (-0.08, 0.21) |
| 10111 | 51 | Meets | Not meets | Meets | Meets | Current | 0.65 | 0.66 (0.62, 0.71) | 0.66 (0.63, 0.69) | 0.01 (-0.16, 0.19) |
|  |  |  |  |  |  |  |  |  |  |  |
| 1012 | 2657 | Not meets | Meets | Not meets | Meets | Never | 0.62 | 0.67 (0.63, 0.70) | 0.66 (0.64, 0.68) | 0.04 (-0.06, 0.15) |
| 1101 | 6179 | Not meets | Meets | Meets | Not meets | Current | 0.61 | 0.67 (0.64, 0.70) | 0.68 (0.66, 0.70) | -0.05 (-0.15, 0.04) |
| 11102 | 8451 | Meets | Meets | Meets | Not meets | Never | 0.63 | 0.67 (0.64, 0.70) | 0.68 (0.66, 0.70) | -0.02 (-0.11, 0.07) |
| 10112 | 598 | Meets | Not meets | Meets | Meets | Never | 0.65 | 0.68 (0.64, 0.72) | 0.67 (0.65, 0.69) | 0.05 (-0.08, 0.19) |
| 11101 | 1433 | Meets | Meets | Meets | Not meets | Current | 0.63 | 0.68 (0.65, 0.72) | 0.67 (0.65, 0.69) | 0.05 (-0.06, 0.17) |
|  |  |  |  |  |  |  |  |  |  |  |
| 10011 | 23 | Meets | Not meets | Not meets | Meets | Current | 0.62 | 0.68 (0.63, 0.73) | 0.68 (0.65, 0.71) | -0.0003 (-0.18, 0.18) |
| 111 | 299 | Not meets | Not meets | Meets | Meets | Current | 0.66 | 0.68 (0.64, 0.72) | 0.67 (0.65, 0.69) | 0.06 (-0.09, 0.21) |
| 10012 | 229 | Meets | Not meets | Not meets | Meets | Never | 0.64 | 0.69 (0.65, 0.73) | 0.69 (0.67, 0.71) | -0.005 (-0.16, 0.15) |
| 1102 | 21076 | Not meets | Meets | Meets | Not meets | Never | 0.65 | 0.70 (0.67, 0.72) | 0.69 (0.67, 0.71) | 0.04 (-0.04, 0.13) |
| 11110 | 2348 | Meets | Meets | Meets | Meets | Previous | 0.65 | 0.70 (0.67, 0.73) | 0.70 (0.68, 0.72) | -0.01 (-0.12, 0.09) |
|  |  |  |  |  |  |  |  |  |  |  |
| 11 | 141 | Not meets | Not meets | Not meets | Meets | Current | 0.68 | 0.70 (0.65, 0.74) | 0.69 (0.67, 0.72) | 0.03 (-0.14, 0.20) |
| 1 | 792 | Not meets | Not meets | Not meets | Not meets | Current | 0.60 | 0.70 (0.66, 0.73) | 0.74 (0.72, 0.76) | -0.20 (-0.34, -0.06) |
| 11002 | 2505 | Meets | Meets | Not meets | Not meets | Never | 0.66 | 0.70 (0.67, 0.73) | 0.70 (0.68, 0.72) | 0.01 (-0.09, 0.12) |
| 12 | 803 | Not meets | Not meets | Not meets | Meets | Never | 0.66 | 0.70 (0.67, 0.74) | 0.70 (0.68, 0.72) | 0.01 (-0.12, 0.15) |
| 11001 | 544 | Meets | Meets | Not meets | Not meets | Current | 0.66 | 0.70 (0.66, 0.74) | 0.69 (0.67, 0.71) | 0.05 (-0.09, 0.19) |
|  |  |  |  |  |  |  |  |  |  |  |
| 10101 | 204 | Meets | Not meets | Meets | Not meets | Current | 0.64 | 0.71 (0.67, 0.75) | 0.71 (0.69, 0.73) | -0.02 (-0.18, 0.14) |
| 11010 | 777 | Meets | Meets | Not meets | Meets | Previous | 0.66 | 0.71 (0.67, 0.74) | 0.72 (0.70, 0.74) | -0.05 (-0.18, 0.08) |
| 1110 | 4471 | Not meets | Meets | Meets | Meets | Previous | 0.67 | 0.71 (0.69, 0.74) | 0.71 (0.69, 0.73) | 0.02 (-0.07, 0.12) |
| 101 | 1603 | Not meets | Not meets | Meets | Not meets | Current | 0.67 | 0.72 (0.69, 0.75) | 0.72 (0.70, 0.74) | 0.01 (-0.10, 0.13) |
| 1002 | 6303 | Not meets | Meets | Not meets | Not meets | Never | 0.69 | 0.73 (0.71, 0.76) | 0.71 (0.69, 0.73) | 0.13 (0.031, 0.22) |
|  |  |  |  |  |  |  |  |  |  |  |
| 110 | 1163 | Not meets | Not meets | Meets | Meets | Previous | 0.68 | 0.73 (0.70, 0.76) | 0.75 (0.73, 0.77) | -0.06 (-0.19, 0.06) |
| 10102 | 1000 | Meets | Not meets | Meets | Not meets | Never | 0.71 | 0.74 (0.70, 0.77) | 0.72 (0.70, 0.74) | 0.09 (-0.03, 0.22) |
| 102 | 4689 | Not meets | Not meets | Meets | Not meets | Never | 0.70 | 0.74 (0.71, 0.76) | 0.73 (0.71, 0.74) | 0.06 (-0.04, 0.16) |
| 10110 | 353 | Meets | Not meets | Meets | Meets | Previous | 0.70 | 0.74 (0.70, 0.77) | 0.74 (0.72, 0.76) | -0.006 (-0.16, 0.15) |
| 10001 | 104 | Meets | Not meets | Not meets | Not meets | Current | 0.76 | 0.74 (0.70, 0.78) | 0.73 (0.71, 0.75) | 0.04 (-0.13, 0.22) |
|  |  |  |  |  |  |  |  |  |  |  |
| 10002 | 357 | Meets | Not meets | Not meets | Not meets | Never | 0.72 | 0.75 (0.71, 0.78) | 0.74 (0.72, 0.76) | 0.03 (-0.11, 0.19) |
| 11100 | 8651 | Meets | Meets | Meets | Not meets | Previous | 0.71 | 0.75 (0.72, 0.77) | 0.75 (0.73, 0.76) | 0.002 (-0.09, 0.09) |
| 1100 | 20101 | Not meets | Meets | Meets | Not meets | Previous | 0.71 | 0.75 (0.72, 0.77) | 0.75 (0.74, 0.77) | -0.03 (-0.12, 0.05) |
| 2 | 1674 | Not meets | Not meets | Not meets | Not meets | Never | 0.71 | 0.75 (0.72, 0.78) | 0.75 (0.73, 0.76) | 0.02 (-0.09, 0.14) |
| 1010 | 1452 | Not meets | Meets | Not meets | Meets | Previous | 0.73 | 0.75 (0.72, 0.78) | 0.73 (0.71, 0.75) | 0.13 (0.010, 0.26) |
|  |  |  |  |  |  |  |  |  |  |  |
| 10010 | 147 | Meets | Not meets | Not meets | Meets | Previous | 0.70 | 0.76 (0.72, 0.79) | 0.76 (0.74, 0.78) | -0.01 (-0.18, 0.15) |
| 11000 | 2712 | Meets | Meets | Not meets | Not meets | Previous | 0.72 | 0.76 (0.73, 0.79) | 0.77 (0.75, 0.78) | -0.01 (-0.13, 0.09) |
| 1000 | 6196 | Not meets | Meets | Not meets | Not meets | Previous | 0.73 | 0.77 (0.75, 0.80) | 0.77 (0.76, 0.79) | 0.006 (-0.09, 0.10) |
| 10 | 445 | Not meets | Not meets | Not meets | Meets | Previous | 0.77 | 0.78 (0.74, 0.81) | 0.77 (0.75, 0.78) | 0.06 (-0.09, 0.22) |
| 10100 | 1139 | Meets | Not meets | Meets | Not meets | Previous | 0.76 | 0.79 (0.76, 0.82) | 0.78 (0.77, 0.80) | 0.04 (-0.09, 0.17) |
|  |  |  |  |  |  |  |  |  |  |  |
| 10000 | 430 | Meets | Not meets | Not meets | Not meets | Previous | 0.73 | 0.79 (0.76, 0.82) | 0.80 (0.78, 0.81) | -0.05 (-0.21, 0.10) |
| 100 | 4456 | Not meets | Not meets | Meets | Not meets | Previous | 0.76 | 0.79 (0.77, 0.81) | 0.79 (0.77, 0.80) | 0.02 (-0.08, 0.12) |
| 0 | 1642 | Not meets | Not meets | Not meets | Not meets | Previous | 0.76 | 0.80 (0.77, 0.82) | 0.80 (0.79, 0.82) | -0.03 (-0.16, 0.09) |

All estimates are from the main effects plus confounders model

References

1. eng, Qi & Kim, Jean & Omiyale, Wemimo & Bešević, Jelena & Conroy, Megan & May, Margaret & Yang, Zuyao & Wong, Samuel & Tsoi, Kelvin & Allen, Naomi & Lacey, Ben. (2022). Raw and Cooked Vegetable Consumption and Risk of Cardiovascular Disease: A Study of 400,000 Adults in UK Biobank. Frontiers in Nutrition. 9. 10.3389/fnut.2022.831470.
2. Alcohol Change UK. *Unit calculator*. https://alcoholchange.org.uk/alcohol-facts/interactive-tools/unit-calculator
3. WHO. (n.d.). *Body mass index (BMI)*. World Health Organization. https://www.who.int/data/gho/data/themes/topics/topic-details/GHO/body-mass-index
